# Spirocyclic Drimanes from the Marine Fungus *Stachybotrys* sp. Strain MF347

**DOI:** 10.3390/md12041924

**Published:** 2014-04-01

**Authors:** Bin Wu, Vanessa Oesker, Jutta Wiese, Susann Malien, Rolf Schmaljohann, Johannes F. Imhoff

**Affiliations:** 1 Kieler Wirkstoff-Zentrum am GEOMAR Helmholtz Zentrum für Ozeanforschung Kiel, Kiel 24105, Germany; E-Mails: wubin@zju.edu.cn (B.W.); vlutz@geomar.de (V.O.); jwiese@geomar.de (J.W.); smalien@geomar.de (S.M.); rschmaljohann@geomar.de (R.S.); 2 Ocean College, Zhejiang University, Hangzhou 310058, China

**Keywords:** spirocyclic drimane, antibiotic, *Stachybotrys*, marine fungus

## Abstract

A novel spirocyclic drimane coupled by two drimane fragment building blocks **2** and a new drimane **1** were identified in mycelia and culture broth of *Stachybotrys* sp. MF347. Their structures were established by spectroscopic means. This is the first example of spirocyclic drimane coupled by a spirodihydrobenzofuranlactam unit and a spirodihydroisobenzofuran unit; and the connecting position being N-C instead of an N and N connecting unit. Strain MF347 produced also the known spirocyclic drimanes stachybocin A (**12**) and stachybocin B (**11**) featured by two sesquiterpene-spirobenzofuran structural units connected by a lysine residue; the known spirocyclic drimanes chartarlactam O (**5**); chartarlactam K (**6**); F1839A (**7**); stachybotrylactam (**8**); stachybotramide (**9**); and 2α-acetoxystachybotrylactam acetate (**10**); as well as ilicicolin B (**13**), a known sesquiterpene. The relative configuration of two known spirobenzofuranlactams (**3** and **4**) was determined. All compounds were subjected to biological activity tests. The spirocyclic drimane **2**, **11**, and **12**, as well as the sesquiterpene **13**, exhibited antibacterial activity against the clinically relevant methicillin-resistant *Staphylococcus aureus* (MRSA).

## 1. Introduction

The fungal genus *Stachybotrys* (class: Sordariomycetes, order: Hypocreales) comprises approximately 100 species [1]. Members of *Stachybotrys* spp. are distributed worldwide and are commonly isolated from soil and various decaying plant substrates. Most species are able to decompose cellulose efficiently. Dangerous toxinogenic isolates of the *Stachybotrys chartarum* complex have gained importance, when colonizing cellulosic substrates in moist indoor environments [2]. *S. chartarum* is reported to be involved in animal and human toxicoses, which are associated with “sick building syndrome” in wet buildings [3,4].

Marine isolates of *Stachybotrys* spp. have been gained from various marine environments as the rhizosphere of mangroves, soil and mud of the intertidal zone, intertidal pools, brackish waters, marine sediments and sponges, marine algae, and sea fans [5,6,7,8,9,10,11,12].

During the course of a study of isolates of *S. chartarum* obtained from various areas around the world, spirocyclic drimanes were discovered as the major class of secondary metabolites produced by this fungus [3]. This type of compound has been reported to be produced by other species of *Stachybotrys* and members of this class are potent immunosuppressants, particularly the dialdehyde derivatives [13]. However, dialdehyde derivatives were rarely isolated from the genus *Stachybotrys*, since dialdehydes are relatively unstable and often undergo conversion to stachybotrylactones [14]. Although many spirocyclic drimanes were reported to be toxins, they showed various biological effects, such as immunosuppressive activity [13], endothelin receptor antagonistic activity [15,16], and tyrosine kinase inhibitory activity [17].

**Chart 1:**
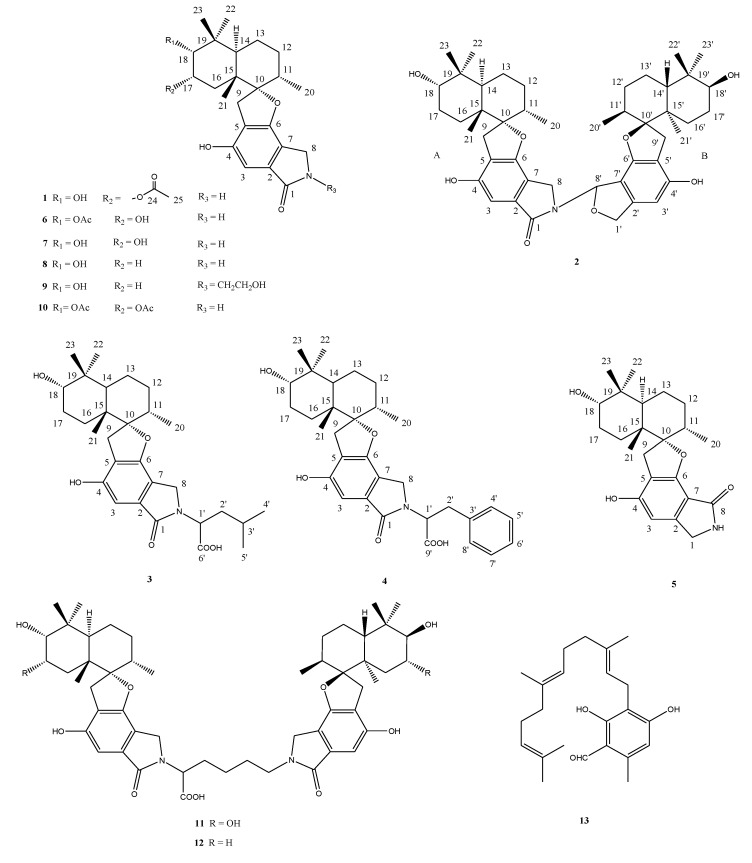
Structures of compounds **1**–**13**.

Fungi from marine habitats, living in a stressful habitat, are of great interest as new promising sources of biologically active products. As marine organisms live in a biologically competitive environment with unique conditions of pH, temperature, pressure, oxygen, light, nutrients, and salinity, the chemical diversity of the secondary metabolites from marine fungi is considerably high [18,19,20]. As far as is known, only two studies focused on biologically active compounds produced by *Stachybotrys* spp. strains originated from marine habitats and identified stachybotrin A and stachybotrin B, as well as a fibrinolytic active compound of unknown structure [8,12]. In order to find new natural products from marine microorganisms exhibiting, e.g., antibacterial and cytototoxic activities, *Stachybotrys* sp. strain MF347 was cultured and the secondary metabolites in the mycelia and the culture broth were investigated. A novel spirocyclic drimane coupled by two drimane fragment building blocks and a new drimane were identified. This is the first example of spirocyclic drimane coupled by a spirodihydrobenzofuranlactam unit and a spirodihydroisobenzofuran unit, and the connecting position being N–C instead of an N and N connecting unit (Chart 1). 

## 2. Results and Discussion

### 2.1. Identification of Strain MF347

**Figure 1:**
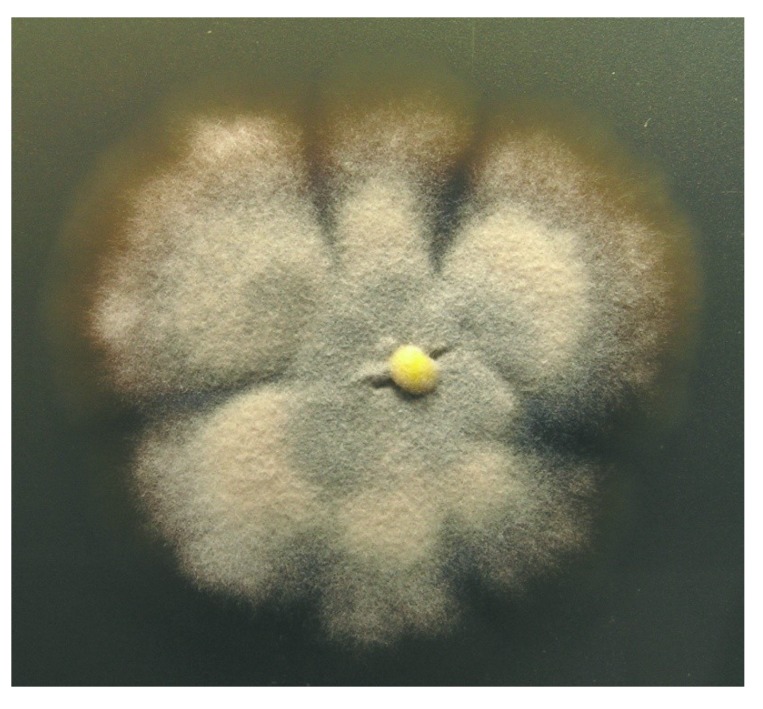
Colony of *Stachybotrys* sp. MF347, grown for 14 days at WSP30 agar.

**Figure 2:**
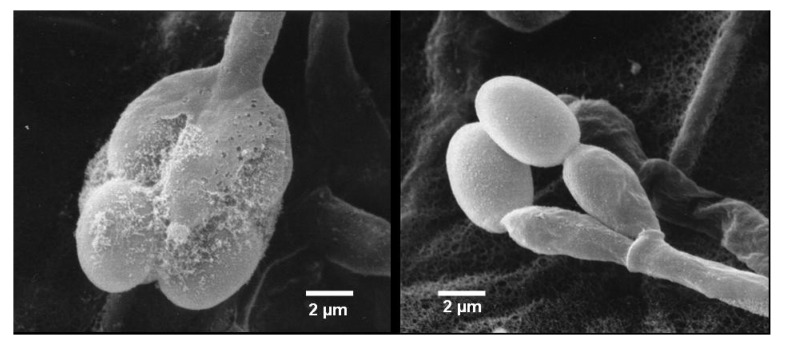
*Stachybotrys* sp. MF347, scanning electron micrograph showing conidiophore with phialides and conidia.

The fungus was isolated by Dr. Karsten Schaumann from a marine driftwood sample and could be taxonomically classified as a *Stachybotrys* species. Colonies growing on WSP30 agar attaining a diameter of 40 mm within 14 days of incubation at 26 °C. Colonies were greyish brown with a brown back side (Figure 1). Ellipsoidal conidia were produced by clusters of phialides in slimy masses at the top of conidiophores (Figure 2). These morphological features are characteristic for the genus *Stachybotrys*, including the well-known species *Stachybotrys chartarum*.

### 2.2. Structural Elucidation

**Figure 3:**
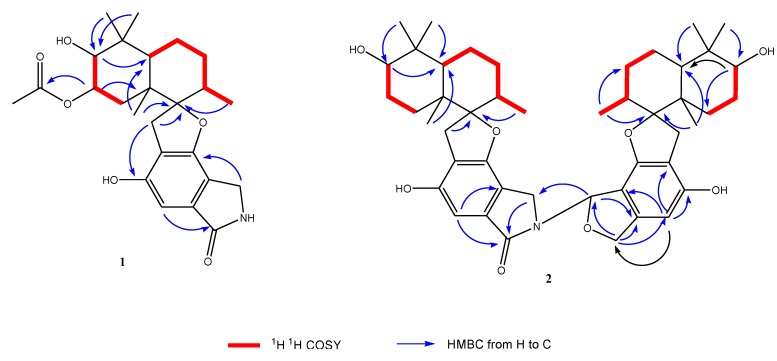
Key ^1^H ^1^H COSY and HMBC correlations of compounds **1 **and **2**.

**Figure 4:**
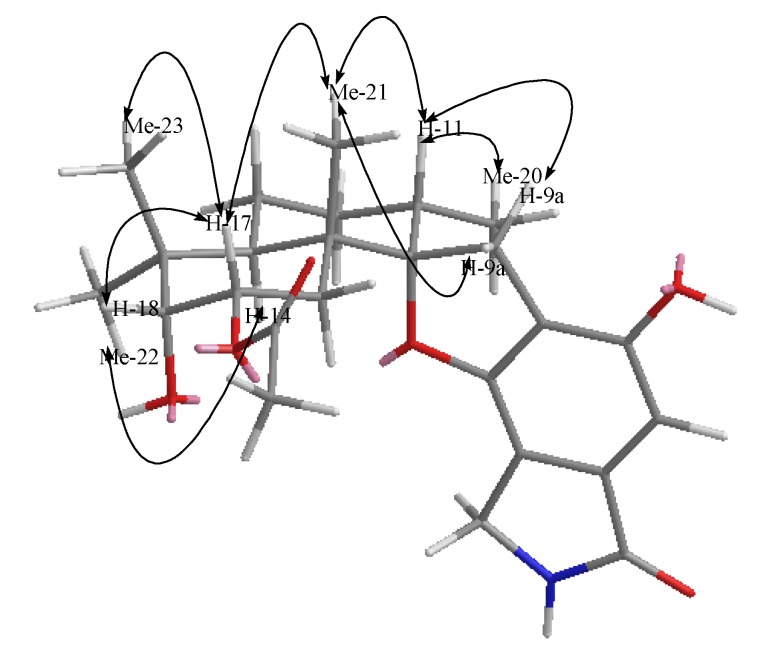
Key NOESY correlations of compound **1**.

Compound **1** was isolated as a yellow powder. The molecular formula was determined to be C_25_H_33_NO_6_ by analysis of the HR-TOF-MS ion peak at *m/z* 466.2181 [M + Na]^+^ (calcd. 466.2200). The IR spectrum suggested the presence of an α,β-unsaturated γ-lactam (1673 cm^−1^) group. ^1^H and ^13^C NMR spectrum (Table 1) showed signals in close agreement with those of the known spirodihydrobenzofuranlactam, F 1839 A (**7**) [21], isolated from the same fungus, except that the acetoxyl group was added in the molecule of **1**. Analysis of the 1D and 2D NMR data (Figure 3) and comparison with those of F 1839 A led to the identification of the planar structure of **1** as drawn [21]. The acetoxyl group was positioned at C-17 from the observation of the HMBC cross peak from the oxygenated proton signal at δ_H_ 5.21 (ddd, *J* = 12.6, 4.4, 2.6 Hz, H-17) to the acetoxyl carbon signal at δ_C_ 172.6 (s, C-24). The 1,3-diaxial NOESY cross peaks of H-17β/Me-23, H-17β/Me-21β, Me-21β/H-11β revealed a 9.14-*trans* ring fusion between two cylcohexyl chair rings of the bicyclic decalin with a β-oriented methyl group at C-15 and α-oriented methyl group at C-11 (Figure 4). The NOESY correlations from the axial methyl proton signals at δ_H_ 1.17 (s, Me-21) to the one of the methylene proton signal at δ_H_ 3.25 (d, *J* = 17.0 Hz, H-9a), and from axial proton signal at δ_H_ 1.91 (m, H-11) to one of the methylene proton signal at δ_H_ 2.93 (d, *J* = 17.0 Hz, H-9b) indicated a β-oriented CH_2_ and an α-oriented oxygen atom bridge at the spirocyclic carbon center of C-10. The diagnostic NOESY correlation from the proton signal at δ_H_ 5.21 (ddd, *J* = 12.6, 4.4, 2.6 Hz, H-17β) to the proton signal at δH 3.48, (d, *J* = 2.1 Hz, H-18β) revealed that the both oxygen at C-17 and C-18 were α-oriented. The relatively small coupling constant between H-17 and H-18 of 2.1 Hz and large coupling constant between H-17 and H-16 of 12.6 Hz confirmed an equatorial β-oriented H-18 and an axial β-oriented H-17 in **1**. Thus, compound **1** was identified as a new spirodihydrobenzofuranlactam, and given the trivial name stachyin A.

**Table 1:** NMR data (500 MHz) for compound **1 **in CD_3_OD.

Position	1	
	δ_C_^a,b^, mult.	δ_H_^c^, mult. (*J* in Hz)
1	174.0, C	-
2	134.9, C	-
3	102.3, CH	6.74, s
4	155.2, C	-
5	118.6, C	-
6	157.5, C	-
7	116.8, C	-
8	43.9, CH_2_	4.47, d (17.4), 4.31, d (17.4)
9	33.1, CH_2_	3.25, d (17.0), 2.93, d (17.0)
10	99.2, C	-
11	38.0, CH	1.91, m
12	32.1, CH_2_	1.66, 1.56, 2 m
13	21.7, CH_2_	1.54, 1.62, 2 m
14	40.8, CH	2.15, m
15	44.8, C	-
16	30.8, CH_2_	1.96, t (12.3), 1.36, dd (12.3, 4.4)
17	72.0, CH	5.21, ddd (12.3, 4.4, 2.1)
18	77.0, CH	3.48, d (2.1)
19	39.9, C	-
20	15.8, CH_3_	0.78, d (6.6)
21	17.3, CH_3_	1.17, s
22	29.1, CH_3_	0.99, s
23	22.5, CH_3_	1.07, s
24	172.6, C	-
25	21.2, C	1.99, s

^a^ Recorded at 125 MHz; ^b^ multiplicities inferred from DEPT and HMQC experiments; ^c^ recorded at 500 MHz.

Compound **2** was obtained as a yellow powder. The HR-TOF-MS exhibited an ion peak at *m/z* 778.4319 [M + Na]^+^ (calcd. 778.4289), corresponding to the molecular formula, C_46_H_61_NO_8_. The characteristic feature of the ^13^C NMR spectrum of **2** is the presence of 15 pairs of identical or almost equivalent carbon signals, which are assigned to two sesquiterpene units. When comparing the NMR data of **1** (Table 2) with those of stachybotrylactam (**8**), compound **2** was deduced to be the coupling of two units of spirodihydrobenzofuranlactam/lactone with one stachybotrylactam substructure. The remaining half of the molecule was proved by detailed analysis of 1D and 2D NMR spectra of **2** to have the same sesquiterpene part in its structure (Figure 3). From the analysis of the molecule formula, C_46_H_61_NO_8_, the remaining unit was deduced to be a spirodihydrobenzofuranlactone or spirodihydrobenzofuran instead of spirodihydrobenzofuranlactam. The presence of the oxygenated methine (δ_C_ 85.8, δ_H_ 7.24) and oxygenated methylene (δ_C_ 74.0, δ_H_ 5.21, 5.00) implied that the structure of **2** possessed a dihydroisobenzofuran unit in one of the two spirocyclic drimane domains. Detailed analyzes of 2D NMR spectra (^1^H ^1^H COSY, HSQC and HMBC) permitted the construction of the two units, and their connection (Figure 3). The HMBC peaks from the proton signal at δ_H_ 7.24 (br d, *J* = 2.1 Hz, H-8′) in the dihydroisobenzofuran unit to the carbon at δ_C_ 44.3 (t, C-8) in the spirodihydrobenzofuranlactam indicated that the linkage position of the two units was from N to C-8 as drawn. The relative configuration of the sesquiterpene parts of both monomers of **2** was deduced from the analysis of the NOESY spectrum to be the same as those in compounds **1** and the known stachybotrylactam (**8**) (see Supplementary Information). Therefore, the structure of this compound was elucidated as a new spirocyclic drimane coupled by two drimane fragment building blocks and given the trivial name stachyin B. Most dimeric spirodihydrobenzofuranlactams isolated from natural resources are those whose the monomers are connected by a N and N connecting unit, such as N–C(OOH)CH_2_CH_2_CH_2_CH_2_–N in compounds **11** and **12** [16]. The structure of **2** is the first example of spirocyclic drimane coupled by a spirodihydrobenzofuranlactam unit and a spirodihydroisobenzofuran unit, and the connecting position being an N–C instead of an N and N connecting unit.

**Table 2:** NMR data (500 MHz) for compound **2** in CD_3_OD.

Position	Unit A		Position	Unit B	
	δ_C_^a,b^, mult.	δ_H_^c^, mult. (*J* in Hz)		δ_C_^a,b^, mult.	δ_H_^c^, mult. (*J* in Hz)
1	171.1, C	-	1′	74.0, CH_2_	5.21 dd (12.2, 2.1), 5.00, d (12.2)
2	134.8, C	-	2′	143.2, C	-
3	102.4, CH	6.77, s	3′	100.2, CH	6.29, s
4	155.3, C	-	4′	156.6, C	-
5	119.3, C	-	5′	113.8, C	-
6	157.6 ^d^, C	-	6′	157.7 ^d^, C	-
7	114.4, C	-	7′	109.1, C	-
8	44.3, CH_2_	4.30, d (16.8), 3.86, d (16.8)	8′	85.8, CH	7.24, br d (2.1)
9	33.0, CH_2_	3.23, d (17.0), 2.85, d (17.0)	9′	32.4, CH_2_	3.15 d (16.2), 2.76 d (16.2)
10	99.6, C	-	10′	99.6, C	-
11	38.5, CH	1.84, m	11′	38.0, CH	1.72, m
12	32.2, CH_2_	1.54, 1.44, overlap	12′	32.0, CH_2_	1.23, 0.80, 2m
13	22.0, CH_2_	1.54, 1.39, overlap	13′	22.0, CH_2_	1.54, 1.39, overlap
14	41.4, CH	2.05, m	14′	41.2, CH	1.99, m
15	43.4, C	-	15′	43.5, C	-
16	25.3, CH_2_	1.80, 1.15, overlap	16′	25.3, CH_2_	1.80, 1.15, overlap
17	26.1 ^e^, CH_2_	1.96, 1.55, overlap	17′	26.0 ^e^, CH_2_	1.96, 1.55, overlap
18	76.5, CH	3.34, overlap	18′	76.7, CH	3.34, overlap
19	38.0, C	-	19′	38.0, C	-
20	16.0, CH_3_	0.67, d (6.5)	20′	16.1, CH_3_	0.49, d (6.5)
21	16.5, CH_3_	1.10, s	21′	16.5, CH_3_	1.02, s
22	29.0 ^f^, CH_3_	0.98, s	22′	28.8 ^f^, CH_3_	0.96, s
23	23.1 ^g^, CH_3_	0.89, s	23′	23.0 ^g^, CH_3_	0.87, s

^a^ Recorded at 125 MHz; ^b^ multiplicities inferred from DEPT and HMQC experiments; ^c^ recorded at 500 MHz; ^d,e,f,g^ interchangeable.

Compounds **3** and **4** were obtained both as yellow powder. From the detailed analyzes of their MS and NMR spectrum, the structures of **3** and **4** were deduced to be the same compounds patented [22]. No names have been given for these two compounds by the authors, but sum formula and a graphic presentation of the structures were shown. However, no relative configuration and detailed NMR data were reported [13]. Here, we reported the NMR data (Table 3), 2D NMR correlations (Figure 3) and the relative configuration (Chart 1) of the two compounds.

Furthermore, six known spirocyclic drimanes, chartarlactam O [23], chartarlactam K [23], F1839A (**7**) [21], stachybotrylactam (**8**) [3], stachybotramide (**9**) [3] and 2α-acetoxystachybotrylactam acetate (**10**) [1], and two known spirocyclic drimanes featured by two sesquiterpene-spirobenzofuran structural units connected by a lysine residue, stachybocin B (**11**) [16] and stachybocin A (**12**) [16], and a known sesquiterpene, ilicicolin B (**13**) [24] were identified by comparison of their spectroscopic data with those reported in the literature [3,16,21,23,24].

**Table 3:** NMR data (500 MHz) for compound **3** and **4** in CD_3_OD.

Position	3		4	
	δ_C_^a,b^, mult.	δ_H_^c^, mult. (*J* in Hz)	δ_C_^a,b^, mult.	δ_H_^c^, mult. (*J* in Hz)
1	171.8, C	-	171.7, C	-
2	134.4, C	-	134.4, C	-
3	102.2, CH	6.72, s	102.1, CH	6.63, s
4	155.2, C	-	155.1, C	-
5	119.2, C	-	119.0, C	-
6	157.6, C	-	157.5, C	-
7	114.6, C	-	114.6, C	-
8	45.7, CH_2_	4.64, d (16.8), 4.30, d (16.8)	46.2, CH_2_	4.58, d (16.8), 4.29, d (16.8)
9	32.2, CH_2_	3.27, d (16.9), 2.88, d (16.9)	32.2, CH_2_	3.24, d (16.9), 2.83, d (16.9)
10	99.8, C	-	99.7, C	-
11	38.5, CH	1.88, m	38.5, CH	1.88, m
12	33.0, CH_2_	1.62, 1.57, 2 m	33.0, CH_2_	1.62, 1.55, 2 m
13	22.1, CH_2_	1.51, 1.59, 2 m	22.1, CH_2_	1.53, 1.59, overlap
14	41.4, CH	2.16, dd (11.7, 2.5)	41.4, CH	2.13, m
15	43.5, C	-	43.4, C	-
16	25.4, CH_2_	1.86, 1.10, 2 m	25.4, CH_2_	1.88, 1.12, 2 m
17	26.0, CH_2_	2.00, 1.56, 2 m	26.0, CH_2_	2.00, 1.55, 2 m
18	76.5, CH	3.37, t (2.5)	76.5, CH	3.36, t (2.5)
19	38.6, C	-	38.6, C	-
20	16.0, CH_3_	0.76, d (6.6)	15.9, CH_3_	0.70, d (6.6)
21	16.6, CH_3_	1.08, s	16.6, CH_3_	1.07, s
22	29.0, CH_3_	1.01, overlap	29.0, CH_3_	1.01, s
23	23.0, CH_3_	0.91, s	23.0, CH_3_	0.92, s
1′	53.0, CH	5.02, dd (11.4, 4.4)	57.0, CH	5.27, dd (11.1, 5.1)
2′	39.4, CH_2_	1.98 overlap	36.6, CH_2_	3.55, dd (14.8, 5.1), 3.26 (11.1, 5.0)
3′	26.3, CH	1.51 overlap	138.7, C	-
4′	21.4, CH_3_	1.01, d (6.9)	129.6, CH	7.28, dd (8.0, 2.1)
5′	23.5, CH_3_	1.02, d (6.9)	129.6, CH	7.25, td (8.0, 2.1)
6′	172.0 ^d^, C	-	127.7, CH	7.17, tt (8.0, 2.1)
7′	-	-	129.6, CH	7.25, td (8.0, 2.1)
8′	-	-	129.6, CH	7.28, dd (8.0, 2.1)
9′	-	-	174.0 ^d^, C	-

^a^ Recorded at 125 MHz; ^b^ multiplicities inferred from DEPT and HMQC experiments; ^c^ recorded at 500 MHz. ^d^ inferred from HMBC.

### 2.3. Biological Activities

Among the isolates, stachyin B (**2**) inhibited the growth of the Gram-positive test strains *Bacillus subtilis*, *Staphylococcus epidermidis* and the methicillin-resistant *Staphylococcus aureus* (MRSA) with IC_50_ values in the range of 1–1.7 µM (Table 4). Stachybocin A (**12**) and B (**11**) exhibited IC_50_ values in the range of 1.8–4.4 µM against the three Gram-positive strains. The activities observed for ilicicolin B (**13**) were in a similar range with IC_50_ values of 0.7–3.2 µM. Chloramphenicol was used as positive control for *B. subtilis* as well as for the human pathogenic strains, *S. epidermidis* and *S. aureus*, and revealed IC_50_ values of 1.45 (±0.13) µM, 1.81 (±0.04) µM, and 2.46 (±0.4) µM, respectively. The results indicate, that the new compound **2** and the known *Stachybotrys* sp. metabolites **11**, **12**, and **13** showed comparable activities with chloramphenicol. Among the antimicrobial active compounds **2**, **11**, **12**, and **13**, only stachyin B (**2**) and ilicicolin (**13**) showed weak cytotoxic activities with IC_50_ values in the range of 13–30 µM. The new compound **2 **exhibited similar IC_50_ values in comparison to the positive control tamoxifen citrate, which revealed an IC_50_ value of 16.45 (±0.15) µM for the mouse fibroblasts cell line NIH-3T3 and of 17.87 (±0.5) µM for the carcinoma cell line HepG2.

**Table 4:** Antibiotic and cytotoxic activities of the compounds **1**–**13**. The IC_50_ values are given in µM. Compounds **1** and **3**–**10** exhibited IC_50_ values >50 µM in all assays.

No.	Name	Antibiotic Activity		Cytotoxic Activity		
		*B. subtilis*	*S. epidermidis*	*S. aureus* MRSA	NIH-3T3	HepG2
2	stachyin B	1.42 (±0.07)	1.02 (±0.09)	1.75 (±0.09)	13.01 (±0.46)	14.27 (±1.54)
11	stachybocin B	1.77 (±0.32)	4.44 (±0.28)	3.94 (±0.53)	>50	>50
12	stachybocin A	2.03 (±0.23)	2.84 (±0.35)	3.71 (±0.22)	>50	>50
13	ilicicolin B	1.06 (±0.11)	3.18 µM (±0.33)	0.74 (±0.12)	30.00 (±1.20)	>50

The Gram-negative test strain *Klebsiella pneumoniae* as well as the fungal strains *Candida albicans*,* Trichophyton rubrum*, and *Septoria tritici* were not inhibited by the compounds **1**–**13**. All compounds exhibited no worth mentioning activities in the enzymatic assays. The IC_50_ values are >50 µM for the acetylcholinesterase and >10 µM for the glycogen-synthase-kinase 3β and phosphodiesterase 4B2.

## 3. Experimental Section

### 3.1. General Experimental Procedures

Optical rotations were recorded on a Perkin Elmer 241 polarimeter (PerkinElmer Inc., Rodgau, Germany). The IR spectra were run on a Perkin Elmer spectrometer (PerkinElmer Inc.) with an ATR unit. ^1^H NMR (500 MHz) and ^13^C NMR (125 MHz) spectra were measured at 25 °C on a Bruker AVANCE DRX 500 NMR spectrometer (Bruker Daltonics, Bremen, Germany) with TMS as internal standard. The signals of the residual solvent protons and the solvent carbons were used as internal references (δ_H_ 3.31 ppm and δ_C_ 49.0 ppm for methanol-*d*_4_). High-resolution mass spectra were acquired on a benchtop time-of-flight spectrometer (micrOTOF II, Bruker Daltonics) with positive electrospray ionization (ESI).

Analytical reversed phase HPLC-UV/MS experiments were performed using a C_18_ column (Phenomenex Onyx Monolithic C18, 100 × 3.00 mm, Phenomenex Inc., Aschaffenburg, Germany) and applying an H_2_O/acetonitrile (ACN) gradient with 0.1% formic acid added to both solvents (gradient: 0 min 5% ACN, 4 min 60% ACN, 6 min 100% ACN; flow 2 mL/min) on a VWR Hitachi Elite LaChrom system (VWR, Darmstadt, Germany) with an L-2450 diode array detector, an L-2130 pump, and an L-2200 autosampler (VWR, Darmstadt, Germany). This HPLC system was coupled to an ESI-ion trap detector with positive ionization (Esquire 4000, Bruker Daltonics) for mass detection.

The preparative HPLC was conducted with a VWR HPLC-UV system (VWR International LaPrep, VWR) equipped with a pump P110, an UV detector P311, a Smartline 3900 autosampler (Knauer, Berlin, Germany), a LABOCOL Vario-2000 fraction collector (LABOMATIC, Weil am Rhein, Germany) and a Phenomenx Gemini-NX column (C18, 10 µ, 110 A, 100 × 50 mm, Phenomenex Inc.) was used. An H_2_O/acetonitrile (ACN) gradient with 0.1% formic acid added to both solvents was applied (gradient: 0 min 30% ACN with a flow of 40 mL/min; 0.5 min 30% ACN, 17.5 min 60% ACN, 22 min 100% ACN, 26 min 30% ACN; flow 100 mL/min).

Semi-preparative HPLC was carried out using a HPLC-UV system (VWR Hitachi Elite LaChrom system, VWR) consisting of an L-1230 pump, an L-2450 diode array detector, an L-2200 autosampler, a Phenomenex Luna column (Silica (2), 5 µ, 100 A, 250 × 10 mm, Phenomenex Inc.) and a Phenomenex Gemini-NX column 1 (C18, 10 µ, 110 A, 100 × 50 mm, Phenomenex Inc.), respectively. The specification of the columns, solvents, and gradients as applied for the purification of the compounds **1**–**13** as well as the respective retension times and yields are listed in Table 5.

**Table 5:** Purification steps and yields of the compounds **1**–**13**.

Compound	Purification Step	Column	Gradient	Flow (mL/min)	UV Detection at (nm)	*t*_R_ (min)	Yield (mg)
**1**	-	NP	0 min 20% C, 20 min 70% C	5	224	8.8	7.6
**2**	1st	RP	0 min 62% B, 20 min 63% B	15	215	6.0	4.5
**-**	2nd	NP	0 min 20% C, 20 min 30% C	5	-	-	-
**3**	1st	RP	0 min 50% B, 20 min 60% B	15	219	9.5	14.6
**4**	1st	NP	0 min 5% C, 20 min 15% C	5	215	14.8	4.3
**-**	2nd	NP	0 min 15% C, 20 min 35% C	5→8	-	-	-
**5**	-	NP	0 min 20% C, 20 min 70% C	5 (8 min)	224	10.7	8.2
**-**	-	-	-	8 (12 min)	-	-	-
**6**	-	NP	0 min 20% C, 20 min 70% C	5	224	7.2	62.9
**7**	-	NP	0 min 30% C, 20 min 60% C	5	218	6.4	19
**8**	-	NP	0 min 15% C, 20 min 20% C	5	215	12	71.0
**9**	-	NP	0 min 20% C, 20 min 70% C	5	215	13.6	10.5
**10**	1st	RP	isocratic: 40% B, 20 min	15	215	10.5	6.7
**-**	2nd	NP	0 min 10% C, 20 min 25% C	5→6	-	-	-
**11**	-	NP	0 min 20% C, 20 min 70% C	5→8	215	12.0	7.9
**12**	1st	RP	0 min 62% B, 20 min 63% B	15	215	8.0	14.6
**-**	2nd	RP	isocratic: 61% B, 20 min	15	-	-	-
**13**	1st	NP	0 min 0% C, 20 min 50% C	5	215	9.4	4.9

B = acetonitrile supplemented with 0.1% formic acid; C = isopropanol supplemented with 0.1% formic acid; NP = Phenomenex Luna column (Silica (2), 5 µ, 100 A, 250 × 10 mm); RP = Phenomenex Gemini-NX column 1 (C18, 10 µ, 110 A, 100 × 50 mm).

### 3.2. I Fungal Material and Cultivation Conditions

A strain of the order Hypocreales named *Stachybotrys* sp. MF347, was isolated from a driftwood sample collected at Helgoland (North Sea, Germany) and cultivated on GPY medium (0.1% glucose, 0.05 peptone, 0.01% yeast extract, and 1.5% agar dissolved in sea water). The strain was cultured on WSP30 agar, a modified Wickerham-medium, which consisted of 1% glucose, 0.5% peptone, 0.3% yeast extract, 0.3% malt extract, 3% sodium chloride, and 1.5% agar (pH = 6.8) [25] to yield cell material for storage. MF347 was stored at the Kultursammlung Mariner Pilze Kiel (KSMP-Kiel) using two methods, liquid nitrogen and the Microbank System at −80 °C (Mast Diagnostika, Reinfeld, Germany). The identification based on morphological criteria was performed using scanning electron microscopy. Young WSP30 agar colonies were cut in 1cm^2^ samples, transferred through an ethanol series (30%, 50%, 70%, 90%, 3 × 100%; every 15 min) and subsequently critical-point-dried in liquid carbon dioxide (Balzers CPD030, Oerlikon Balzers Coating Germany GmbH, Bingen am Rhein, Germany). Samples were sputter-coated with gold-palladium (Balzers SCD004, Oerlikon Balzers Coating Germany GmbH) and analyzed with a ZEISS DSM 940 (ZEISS, Oberkochen, Germany) scanning electron microscope.

### 3.3. Fermentation, Extraction and Isolation of the Compounds

Strain MF347 was cultured on WSP30 agar plates at 22 °C for 28 days. This pre-culture was used for the inoculation of 12 × 2 L Erlenmeyer flasks containing 750 mL WSP30TM medium (1% glucose, 0.5% peptone, 0.3% yeast extract, 0.3% malt extract, 3% tropic marine salt (pH = 6.8)) each. After incubation for 22 days at 28 °C in the dark as static cultures, extracts of the cultures were obtained. The mycelium was separated from the culture broth. 150 mL ethanol were added to the mycelium of each flask and homogenized. After a centrifugation step at 10,000 rpm for 10 min, the ethanol was removed by evaporation. The remaining aquatic phases of all 12 flasks were combined and extracted twice with 100 mL ethyl acetate. The organic phase was used for evaporation. The resulting residue was dissolved in 20 mL methanol to get the extract of the mycelium. The fermentation broth was extracted with ethyl acetate (400 mL per each flask). 100 mL deionized water were added to the organic phase. The upper phases of all flasks were combined and used for evaporation. The residue was dissolved in 5 mL methanol to get the extract of the culture broth. Both extracts were subjected to analytical HPLC-UV/MS. For the purification of the compounds **1**–**13**, both extracts were combined.

By preparative HPLC 14 fractions were collected. 10 fractions were purified using semi-preparative HPLC. The specification of the columns, solvents, and gradients as applied for the purification of the compounds **1**–**13** as well as the respective retention times and yields are listed in Table 5.

Stachyin A (**1**): yellow powder; [α]^24^_D_ −15° (*c* 0.1, MeOH); UV (MeOH) λ_max_ (log *є*) 218 (4.03), 262 (3.91), 297 (4.12) nm; IR *ν*_max_ 3288, 1673, 1463, 1330, 1252, 1082, 1008, 988, 880, 771 cm^−1^; ^1^H NMR and ^13^C NMR, see Table 1; ESIMS *m/z* 466 [M + Na]^+^; HR-FTICRMS *m/*z 466.2181 [M + Na]^+^ (calcd. for C_25_H_33_NO_6_Na, 466.2200).

Stachyin B (**2**): yellow powder; [α]^24^_D_ −45° (*c* 0.1, MeOH); UV (MeOH) λ_max_ (log *є*) 218 (3.95), 260 (3.90), 302 (4.01) nm; IR *ν*_max_ 2969, 1675, 1609, 1461, 1390, 1350, 1078, 1068, 940, 737 cm^−1^; ^1^H NMR and ^13^C NMR, see Table 2; ESIMS *m/z* 778 [M + Na]^+^; HR-TOF-MS *m/*z 778.4319 [M + Na]^+^ (calcd. for C_46_H_61_NO_8_Na, 778.4289).

### 3.4. Biological Activities Assays

The antimicrobial activities of **1**–**13** against the bacterium *Bacillus subtilis* (DSM 347) and the human pathogenic yeast *Candida albicans* (DSM 1386) were determined according to Ohlendorf *et al*., 2012 [26].The bioassays using the clinically relevant bacterial strains *Staphylococcus epidermidis* (DSM 20044), the methicillin-resistant *Staphylococcus* *aureus* (MRSA) (DSM 18827), and *Klebsiella pneumoniae* DSM 30104 were performed as described by Silber *et al*., 2013 [27]. *S. aureus* and *K. pneumoniae* were investigated in the same manner as *S. epidermidis. Trichophyton rubrum*, a dermatophyte, and the phytopathogenic fungus *Septoria tritici* were tested according to Jansen *et al*., 2013 [28]. The inhibitory activity against phosphodiesterase (PDE-4B2) and the cytotoxic activity against HepG2 (human hepatocellular liver carcinoma cell line) and NIH-3T3 (mouse fibroblasts cell line) were determined according to Schulz *et al*., 2011 [29], apart from the cell concentration with 10,000 HepG2 cells per vial. The determination of the acetylcholinesterase (AchE) inhibitory activity was performed according to Ohlendorf *et al*., 2012 [26]. The glycogen synthase kinase-3β (GSK-3β) inhibition was tested as described by Baki *et al*., 2007 [30]. The concentration of the compounds used in the initial bioassays was 100 µM (antibiotic tests), 50 µM (cytotoxic tests), and 10 µM (enzymatic tests), respectively. To determine the IC_50_ values concentrations of the compounds ranging from 0.1–50 µM were analyzed twice in duplicates. Positive controls were carried out with chloramphenicol (bacteria), nystatin (yeast), clotrimazole (fungi), tamoxifen citrate (cell lines), rolipram (PDE-4B2), huperzine (AchE), and TDZD-8 (GSK-3β).

## 4. Conclusions

Although many compounds isolates from the genus *Stachybotrys* were reported to be toxins [3,4], they showed various biological effects, such as immunosuppressive activity [13] and antihyperlipidemic [23]. Antibiotic activities of spirodihydrobenzofuranlactam and spirodihydrobenzofuranlactone were rarely reported. The spirocyclic drimanes with two sesquiterpene-spirobenzofuran structural units **2**, **11**, and **12** showed antibacterial activity against the clinically relevant methicillin-resistant *Staphylococcus aureus* (MRSA), whereas spirocyclic drimanes with one sesquiterpene-spirobenzofuran structural unit **1**, **3**–**10** exhibit no activities. It is tentatively implied that the structural feature of two sesquiterpene-spirobenzofuran units with either a N-C or a N-N linkage of spirocyclic drimanes is important for antibiotic activity. This is the first example of spirocyclic drimane coupled by a spirodihydrobenzofuranlactam unit and a spirodihydroisobenzofuran unit, and the connecting position being N-C. Stachyin A (**1**) and B (**2**) are structurally interesting, which would provide opportunities to design and synthesize new analogs that could improve the antibiotic and cytotoxic activities of these new compounds.

## Supplementary Files

Supplementary File 1 Supplementary Information (PDF, 3055 KB)
