# Supplementary material for: Spirocyclic Drimanes from the Marine Fungus Stachybotrys sp. Strain MF347
Source: Mar Drugs. 2014 Apr 1;12(4):1924–38. doi: 10.3390/md12041924 (PMC4012445; doi:10.3390/md12041924)
Supplement: Supplementary File 1 — Supplementary Information (PDF, 3055 KB) [file marinedrugs-12-01924-s001.pdf]

Supplementary Information

Figure S1. ^{1}H NMR in CD_3OD for compound **1**.

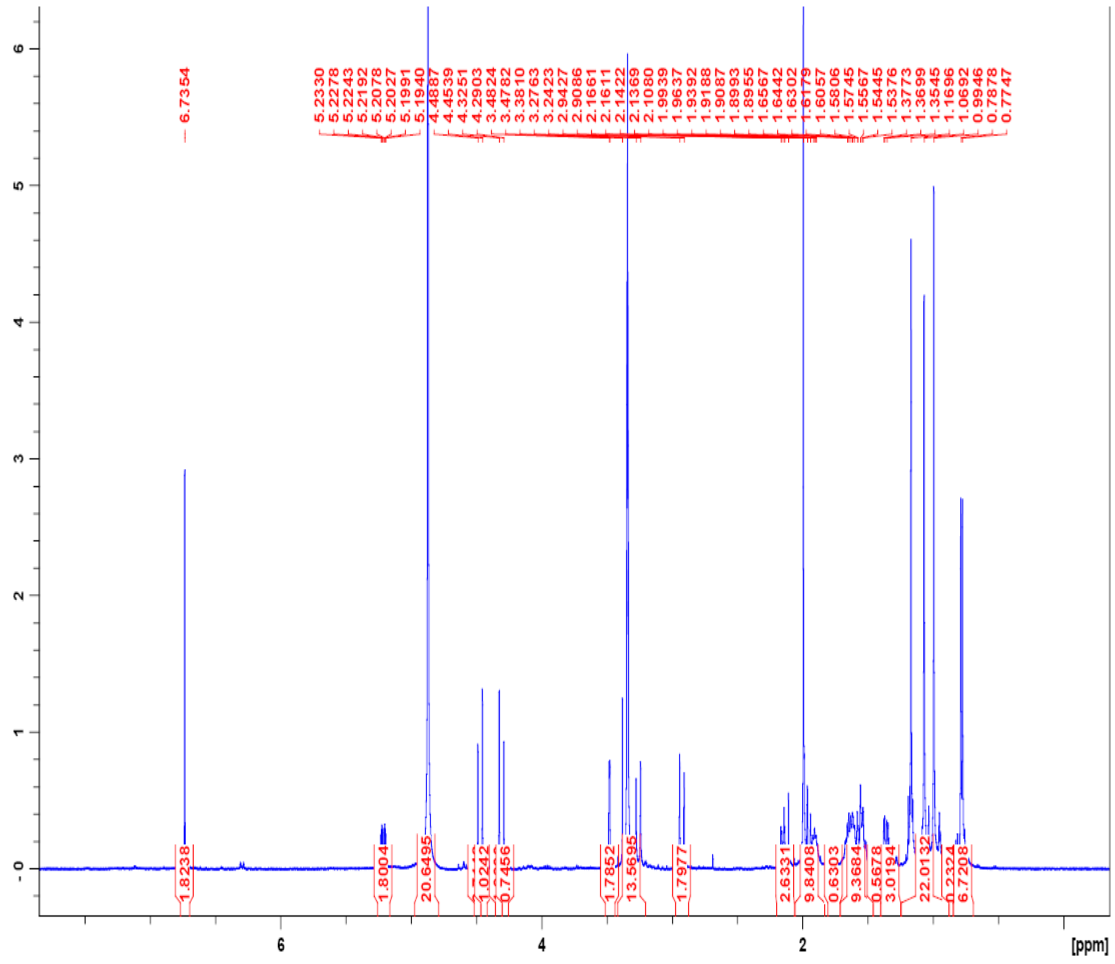

Figure S2. ^{1}H NMR-2 in CD_3OD for compound **1**.

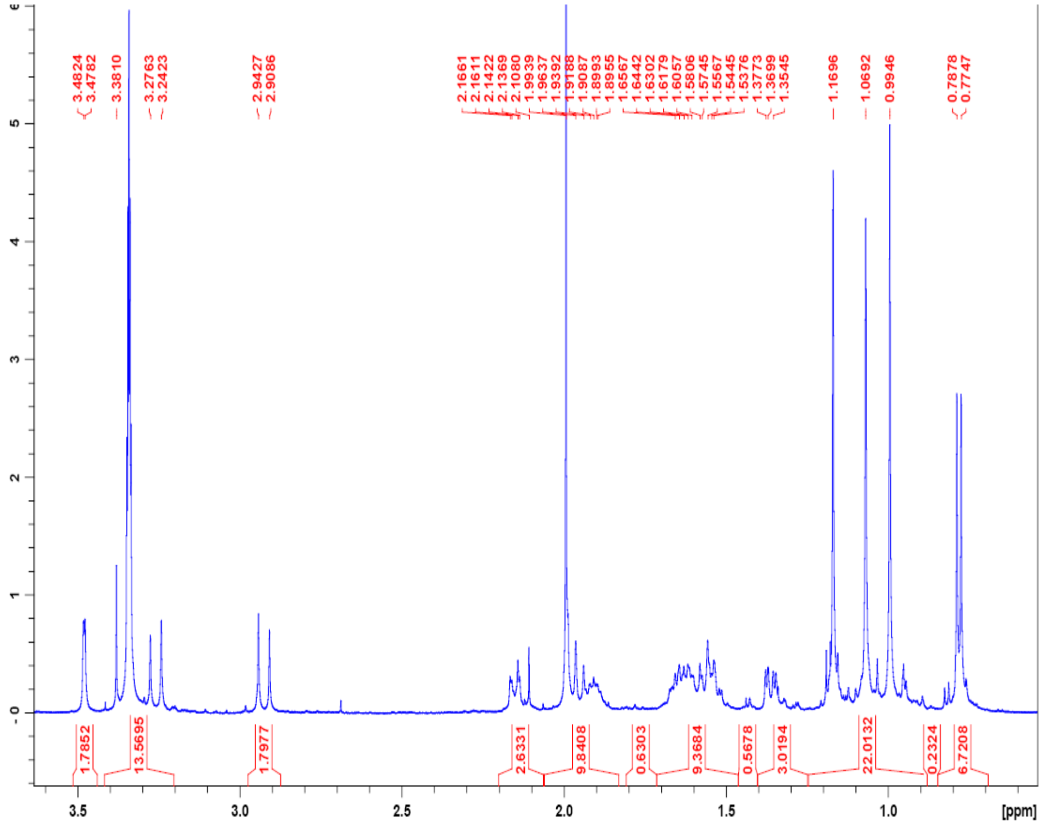

Figure S3. ^{13}C NMR in CD_3OD for compound 1.

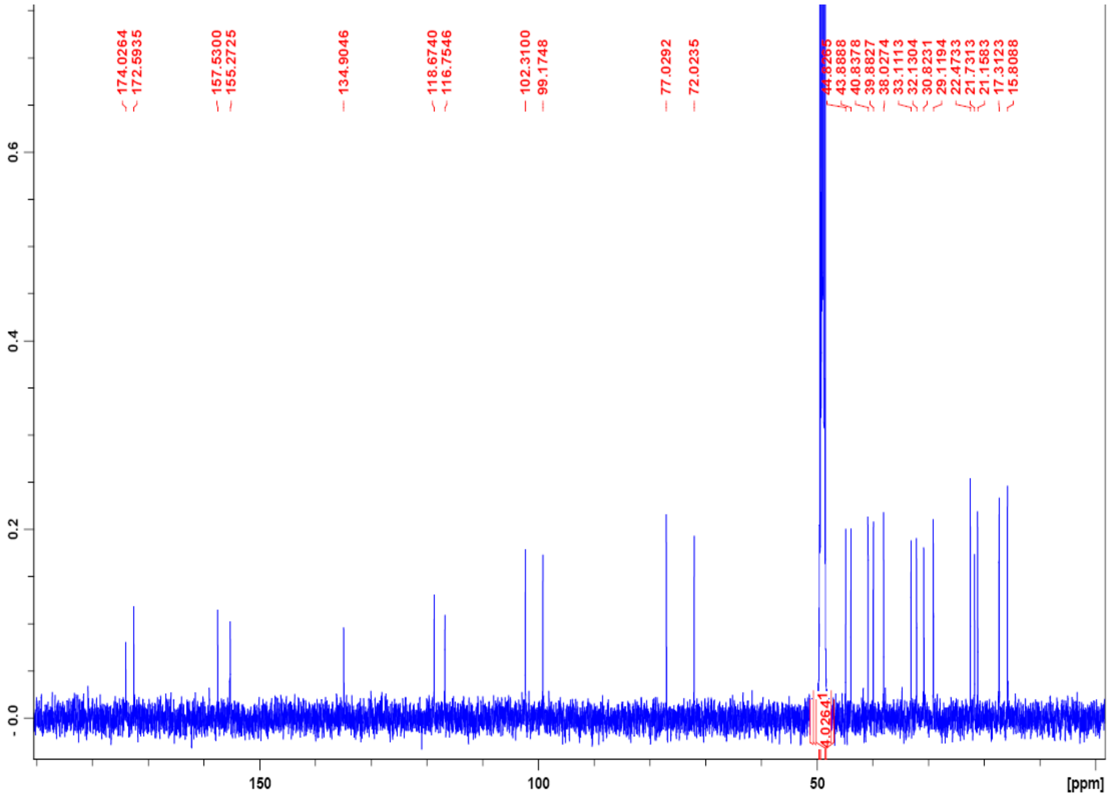

Figure S4. COSY in CD_3OD for compound 1.

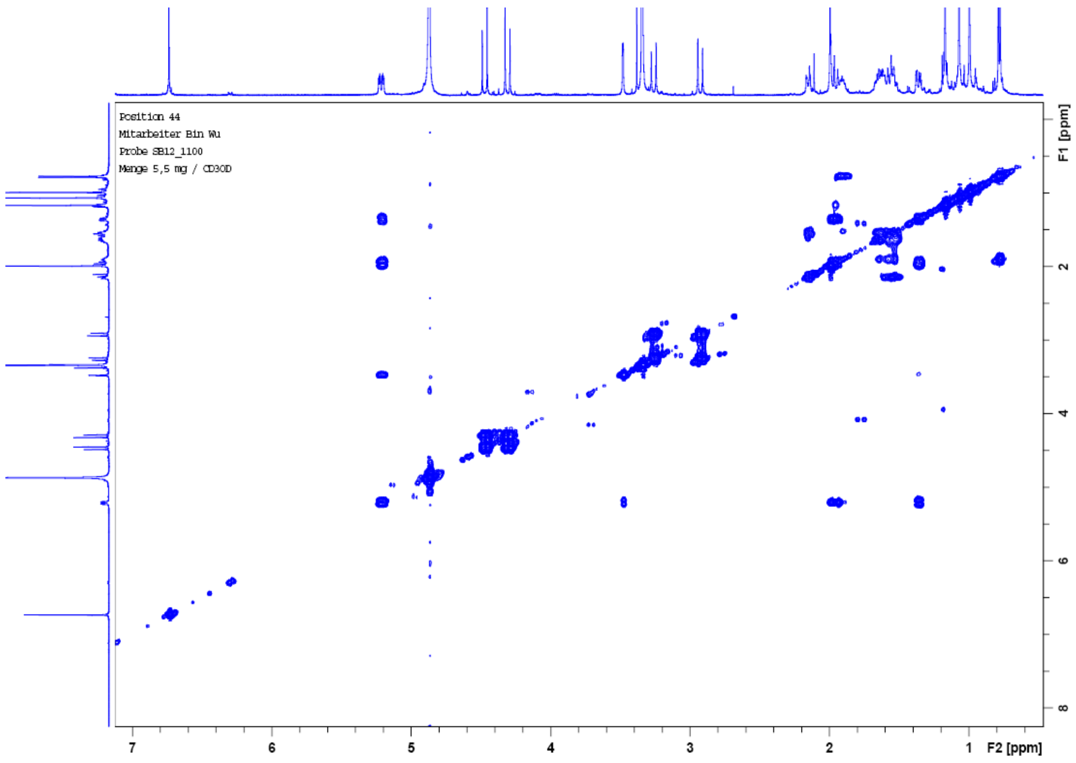

Figure S5. COSY-2 in CD_3OD for compound 1.

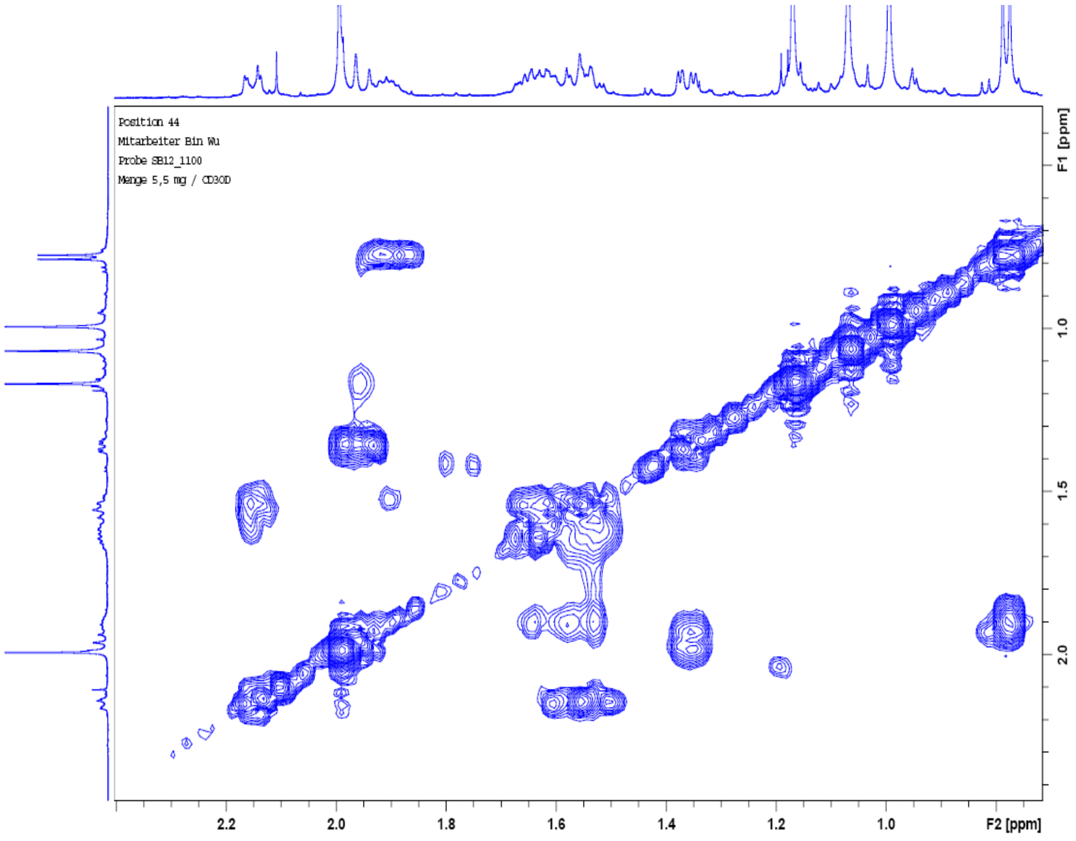

Figure S6. HSQC in CD_3OD for compound **1**.

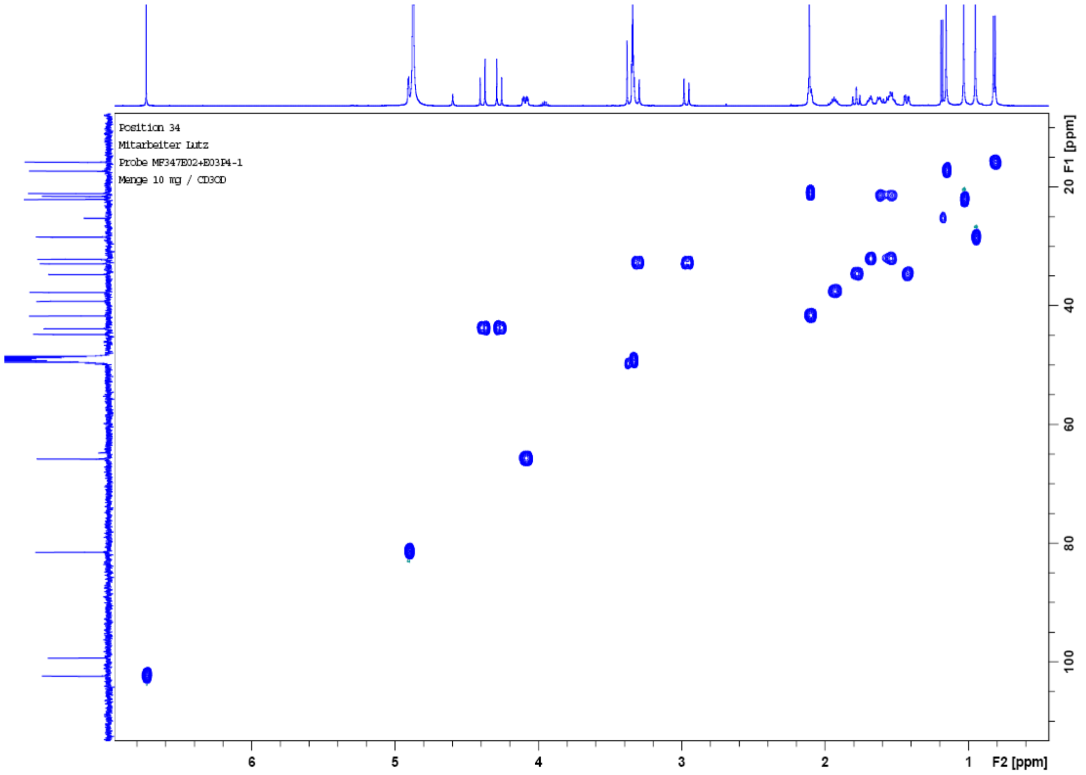

Figure S7. HMBC in CD_3OD for compound **1**.

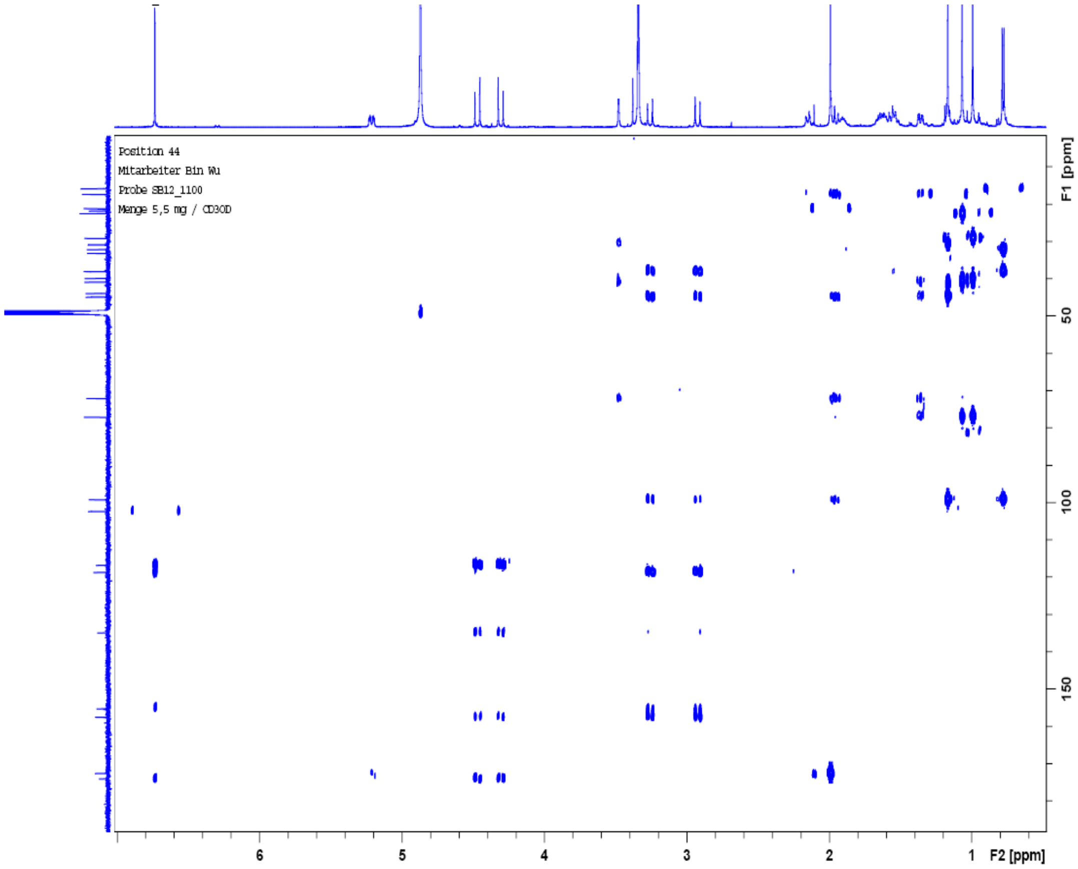

Figure S8. HMBC-2 in CD_3OD for compound **1**.

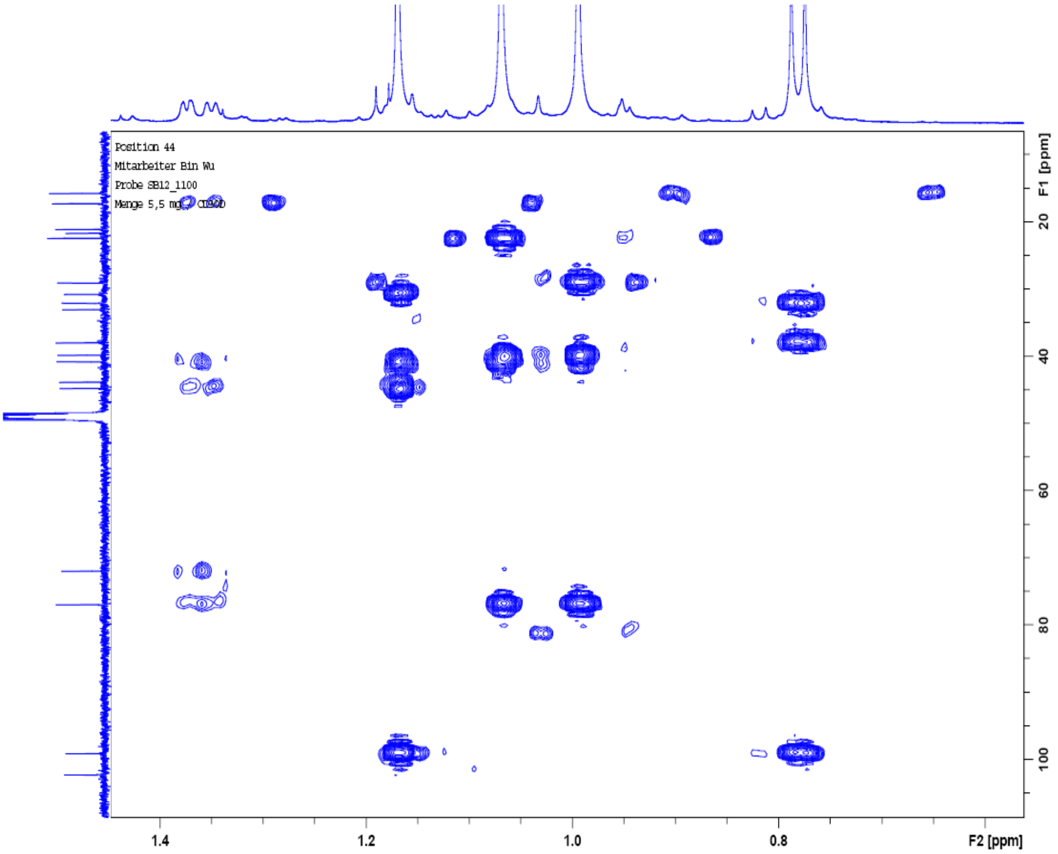

Figure S9. NOESY in CD_3OD for compound **1**.

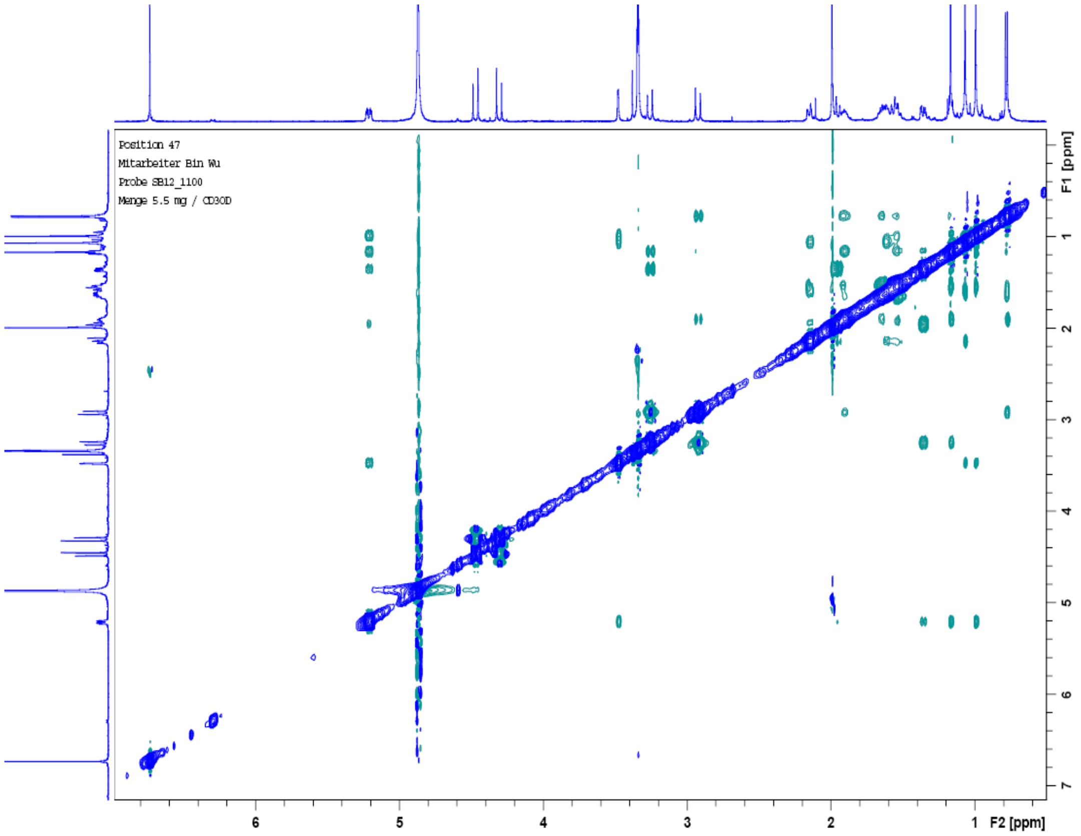

Figure S10. ^{1}H NMR in CD_3OD for compound **2**.

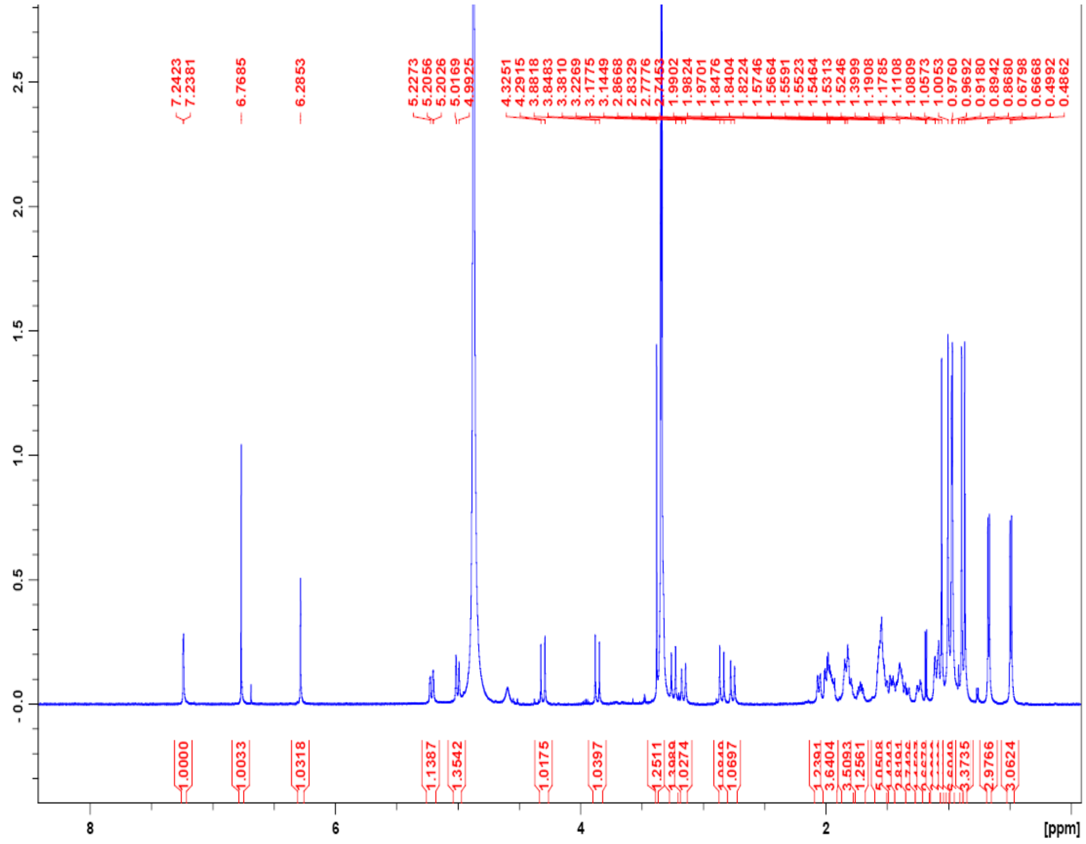

Figure S11. ^{1}H NMR-2 in CD_3OD for compound **2**.

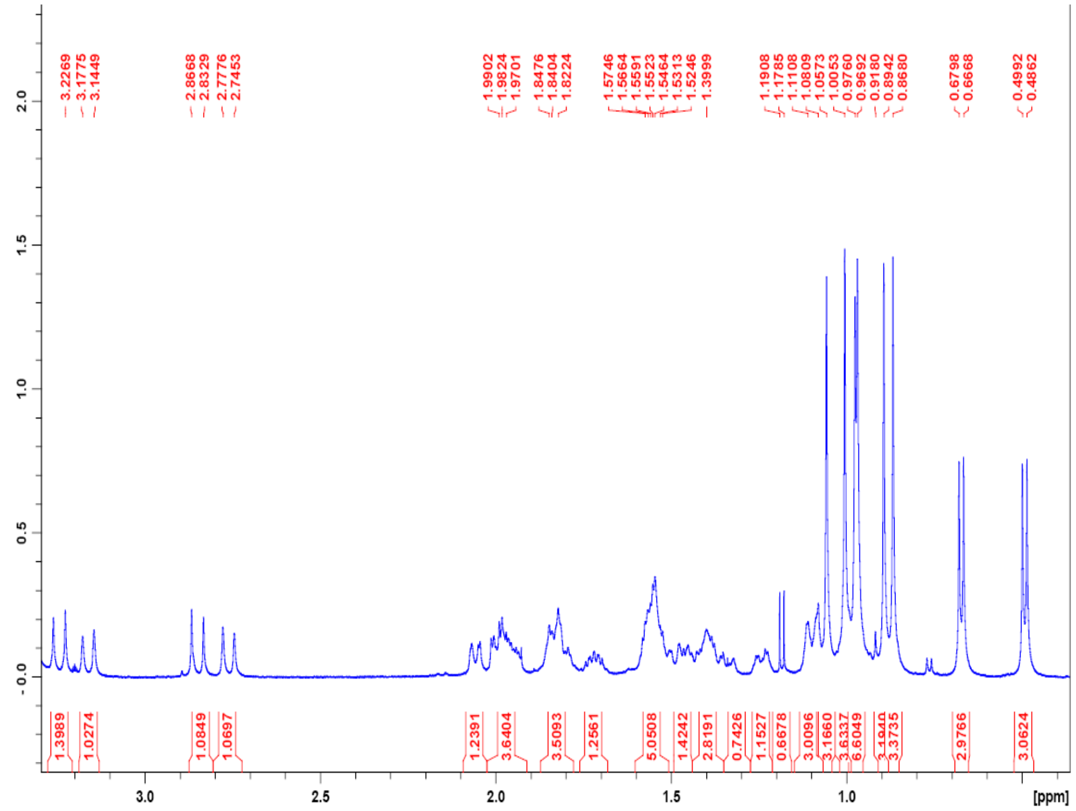

Figure S12. ^{13}C NMR in CD_3OD for compound **2**.

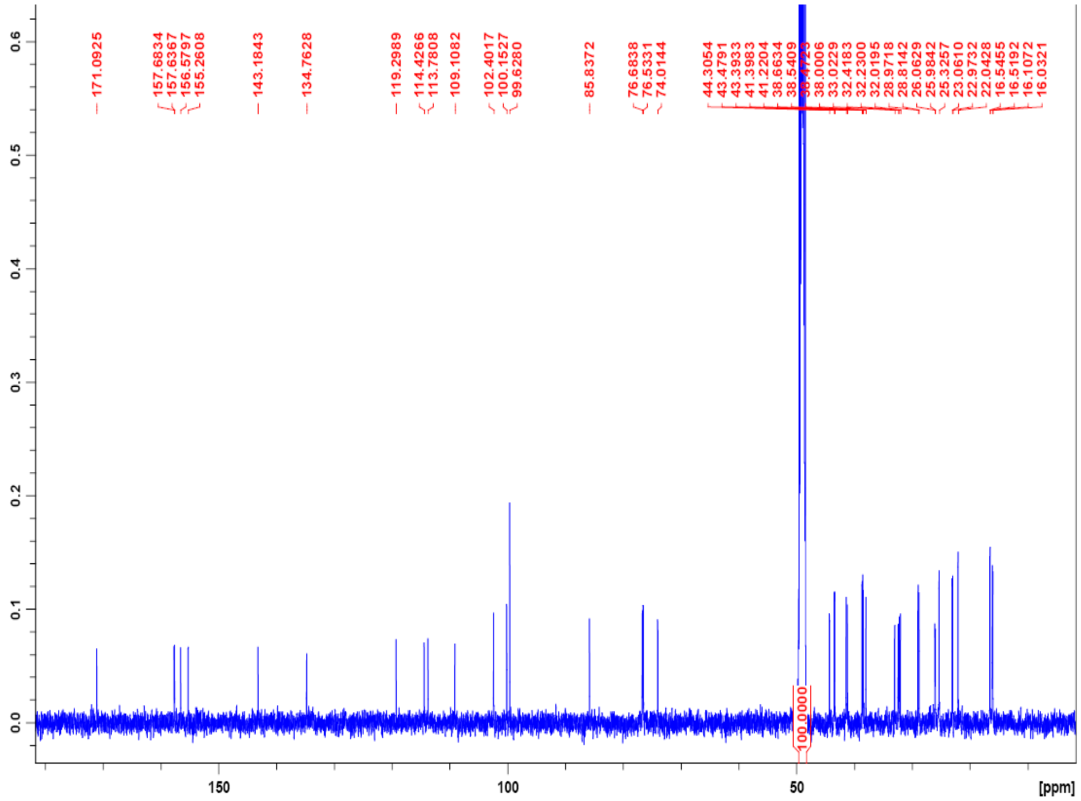

Figure S13. COSY in CD_3OD for compound **2**.

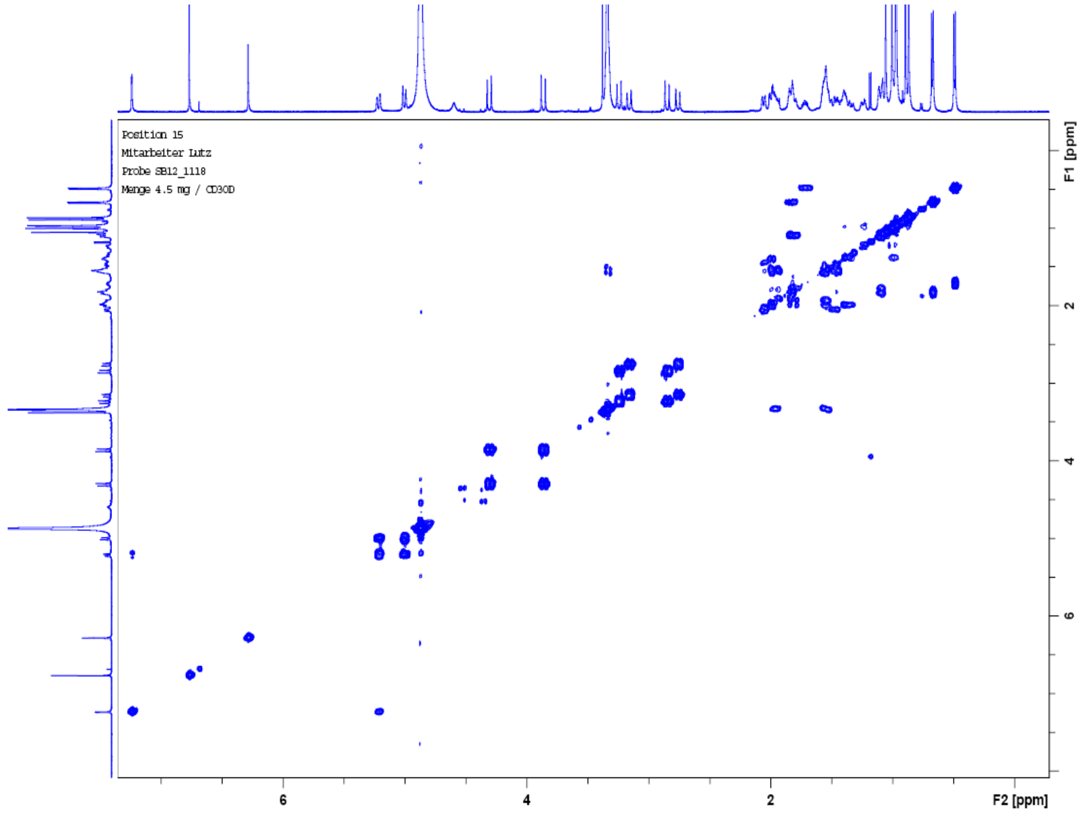

Figure S14. COSY-2 in CD_3OD for compound **2**.

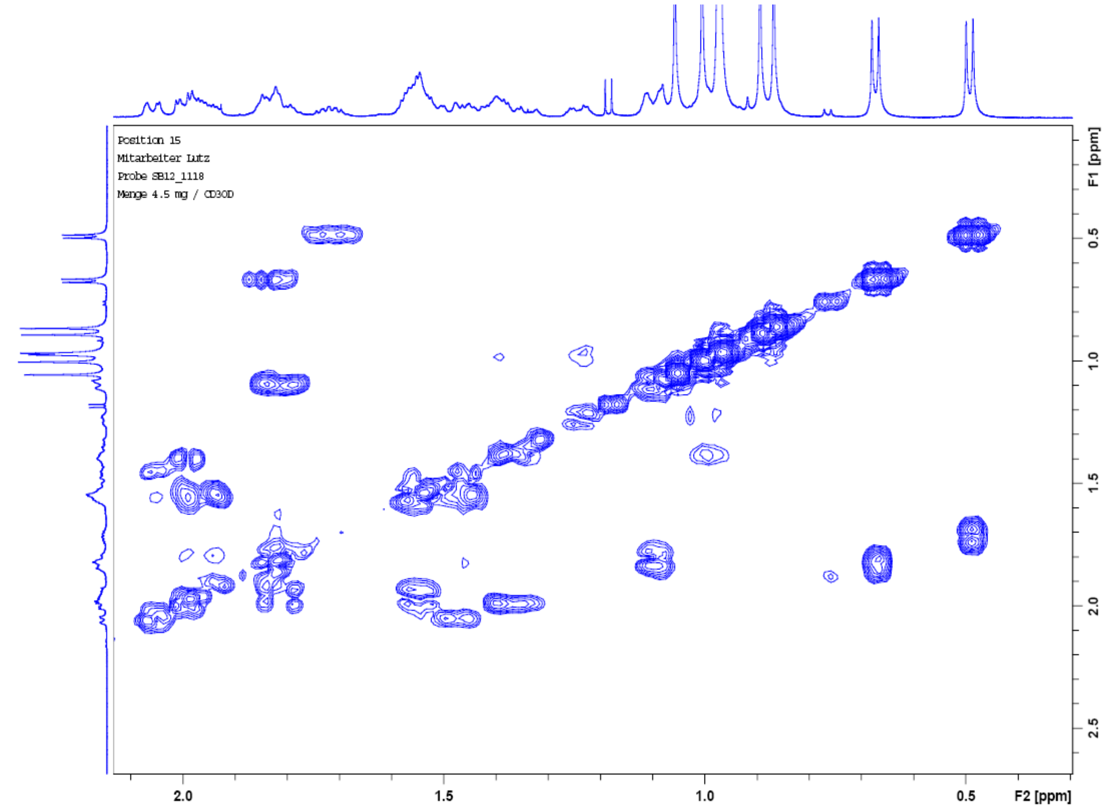

Figure S15. HSQC in CD_3OD for compound **2**.

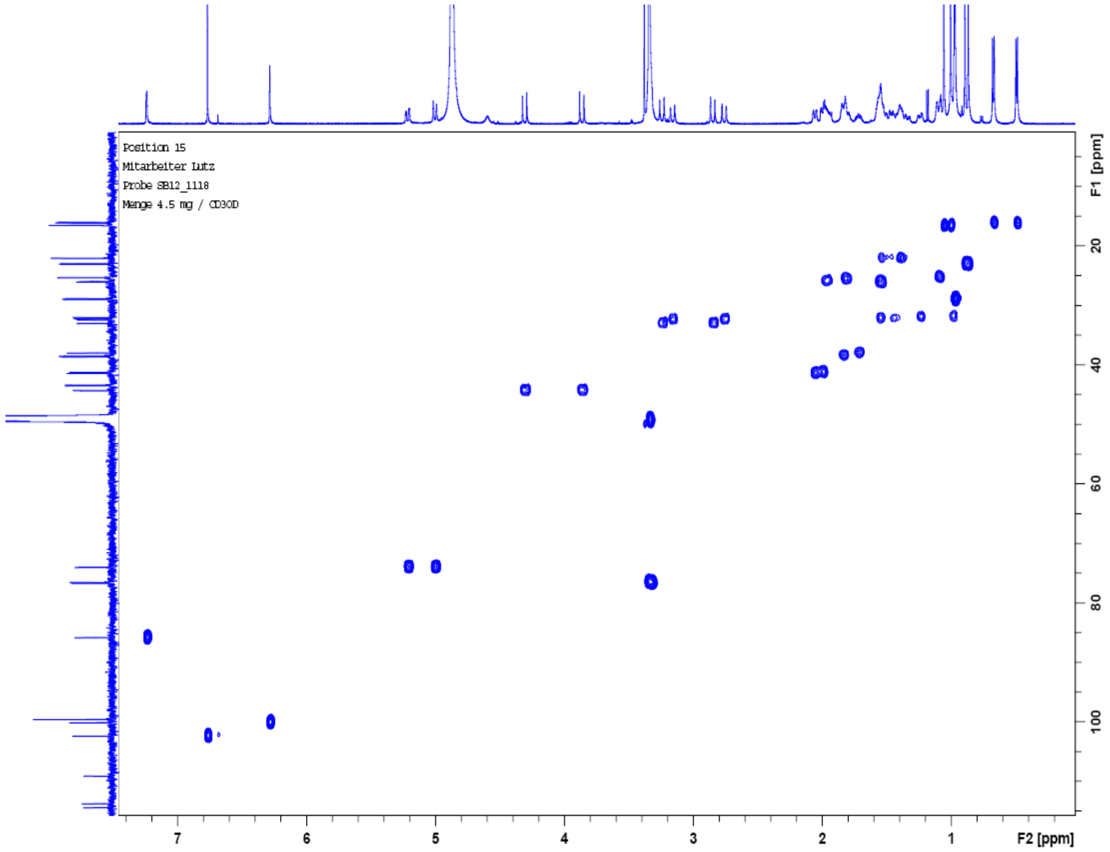

Figure S16. HMBC in CD_3OD for compound **2**.

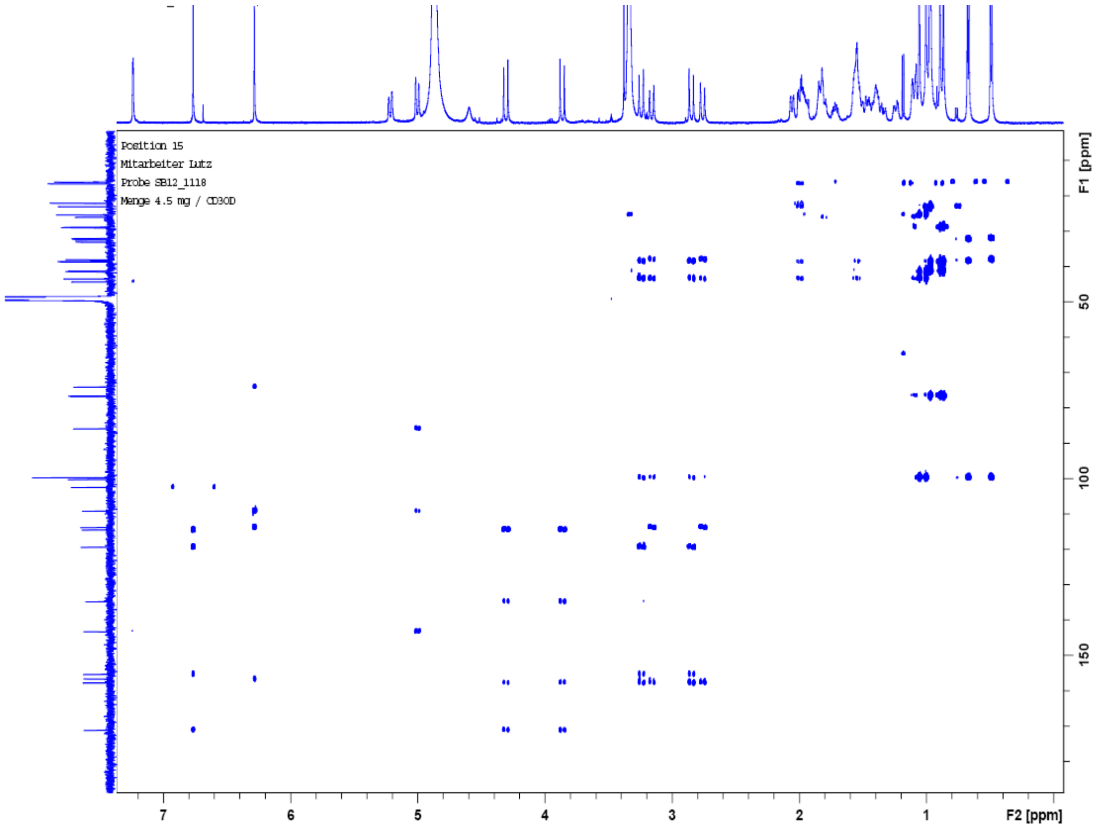

Figure S17. HMBC-2 in CD_3OD for compound **2**.

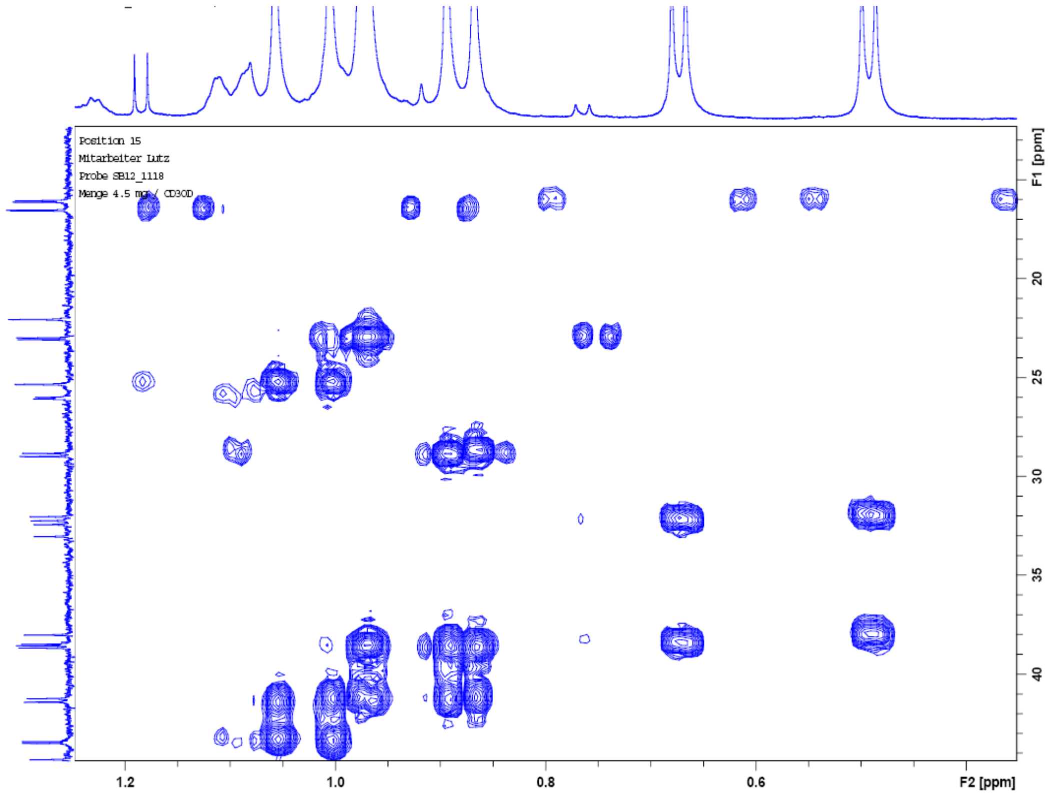

Figure S18. NOESY in CD_3OD for compound **2**.

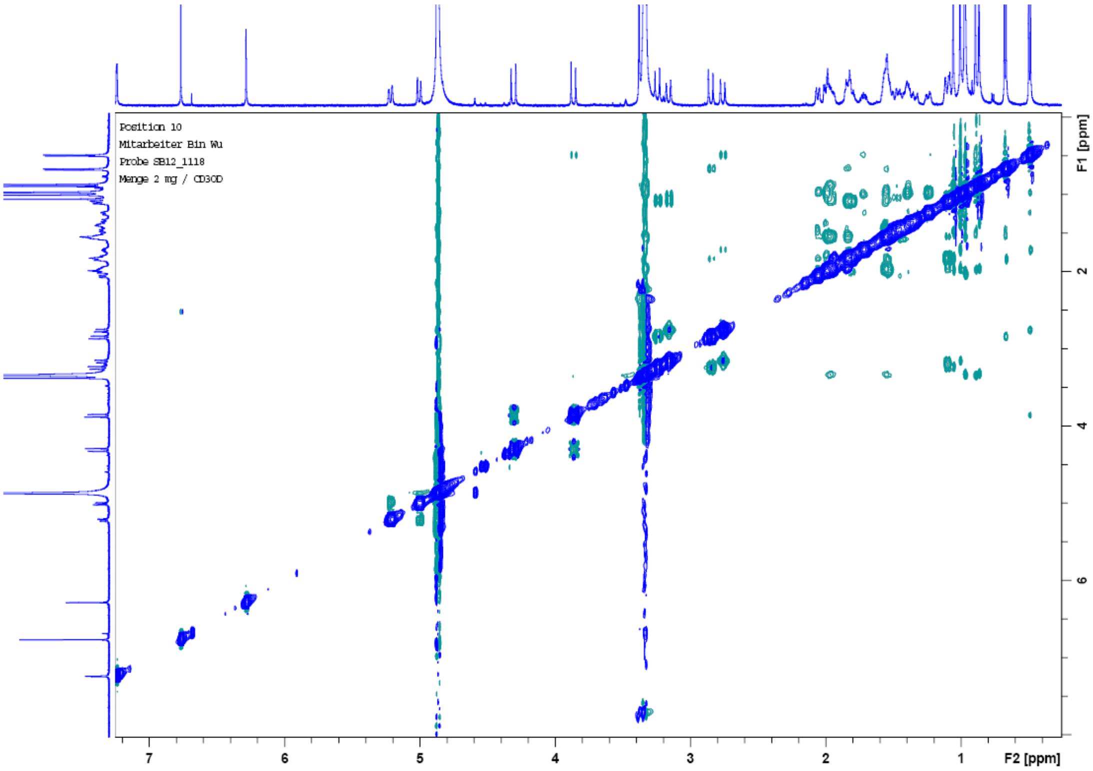

Figure S19. ^{1}H NMR in CD_3OD for compound **3**.

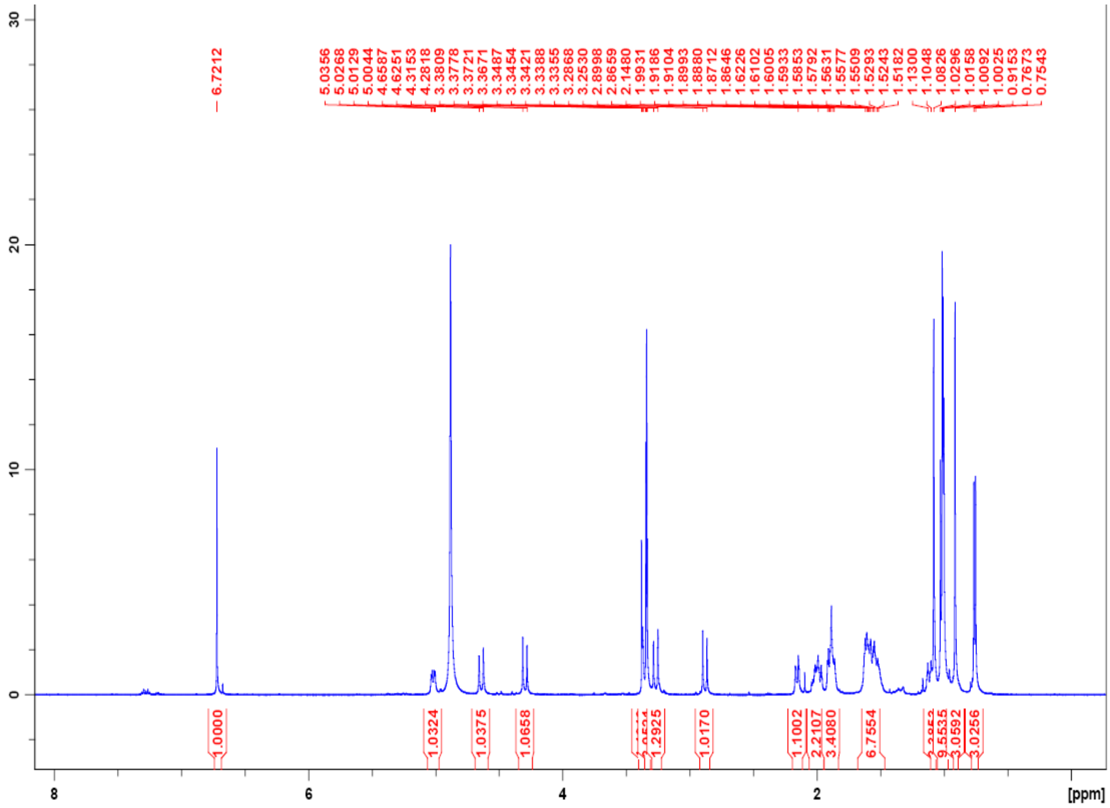

Figure S20. ^{13}C NMR in CD_3OD for compound **3**.

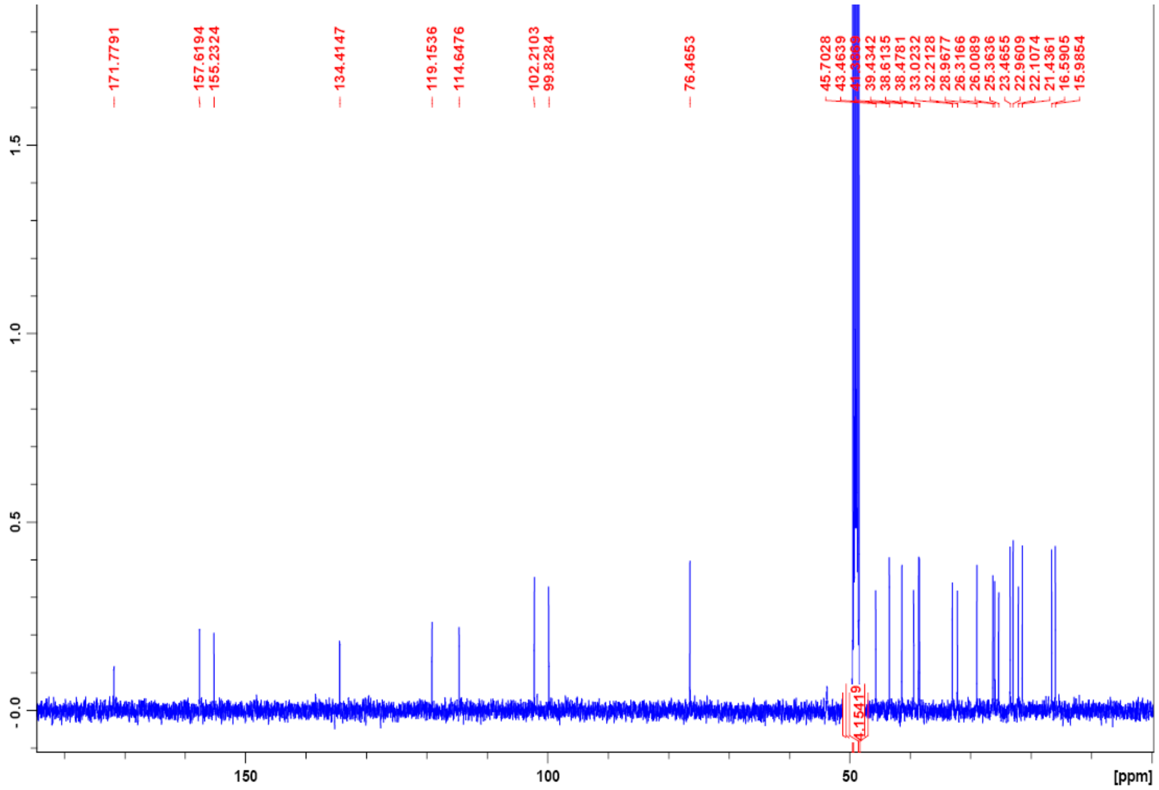

Figure S21. COSY in CD_3OD for compound 3.

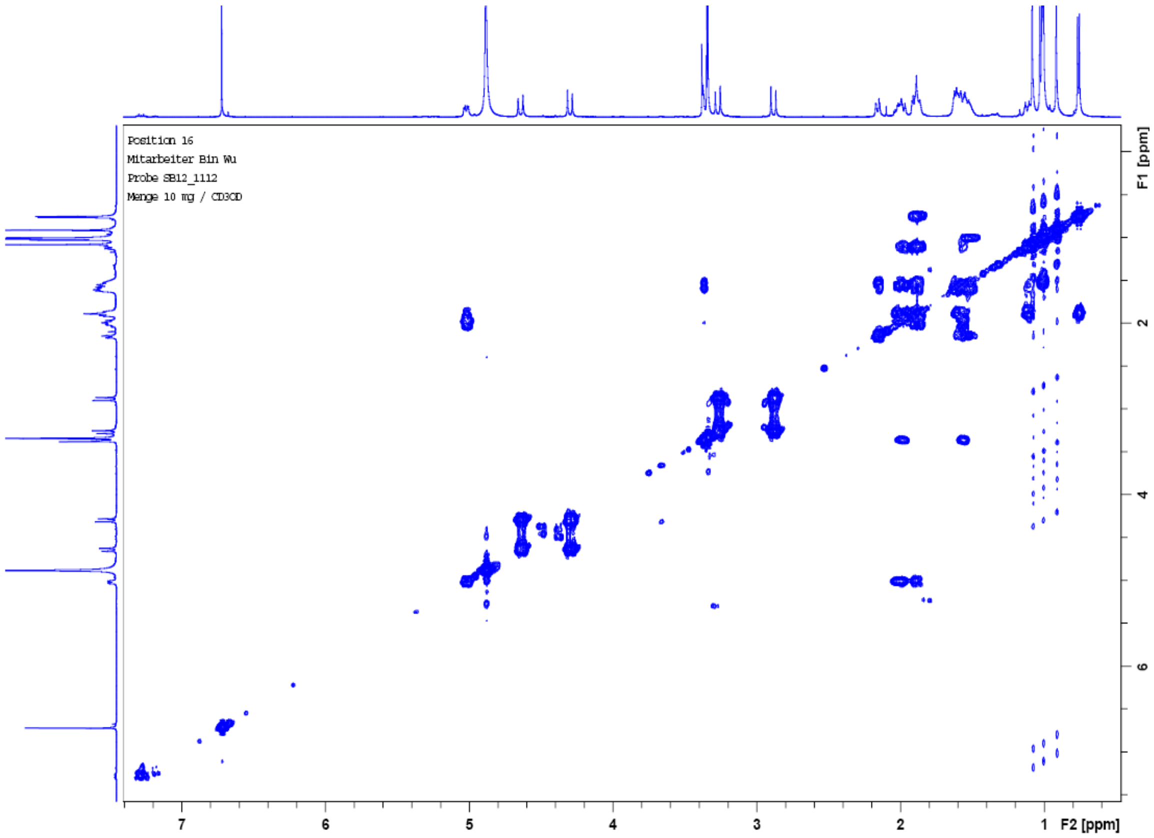

Figure S22. COSY-2 in CD_3OD for compound **3**.

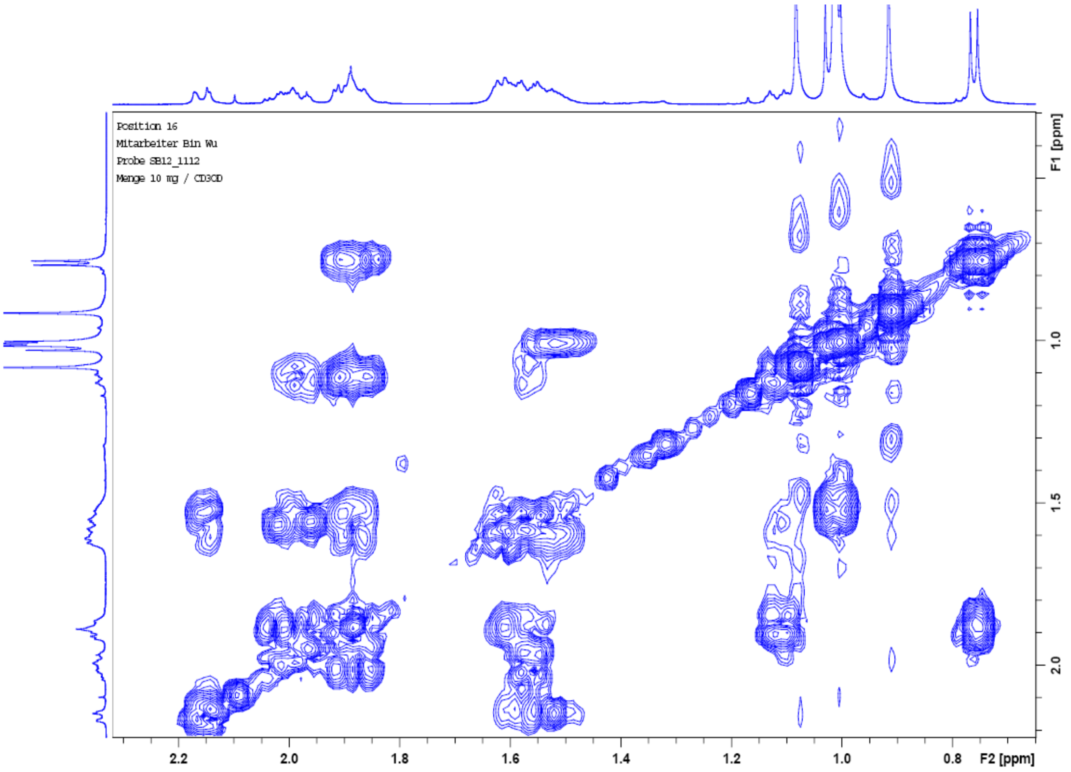

Figure S24. HSQC in CD_3OD for compound **3**.

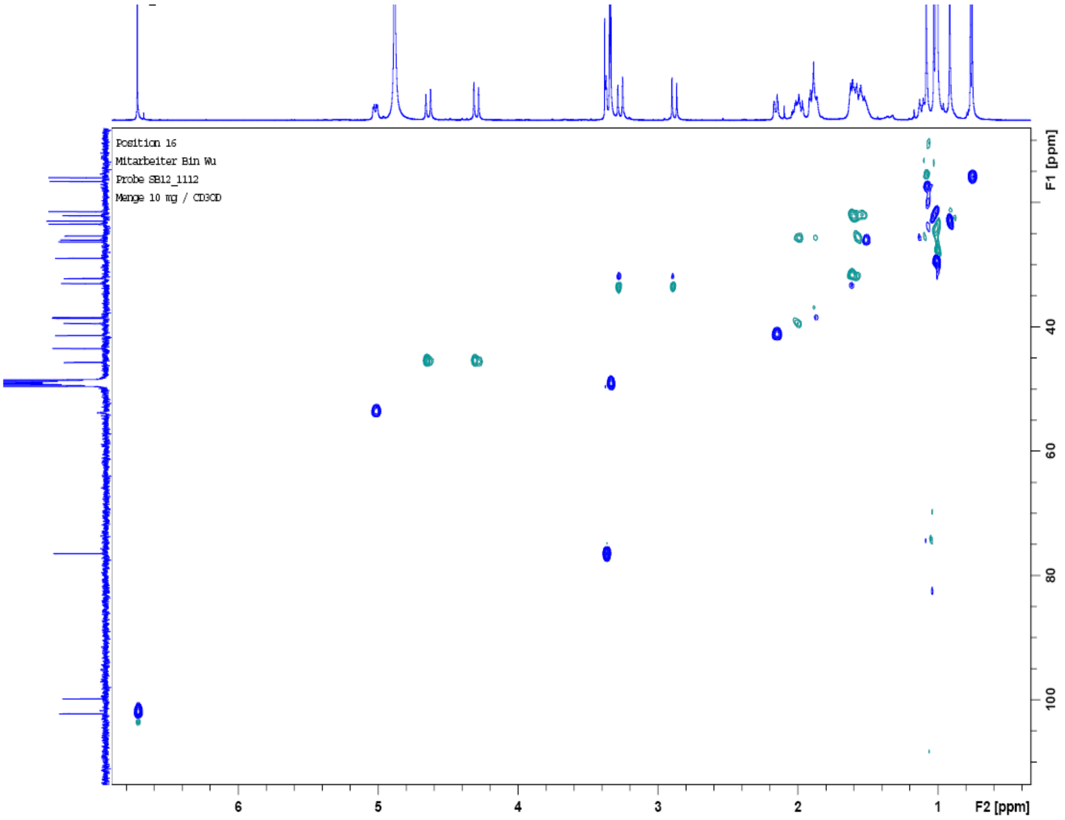

Figure S25. HMBC in CD_3OD for compound 3.

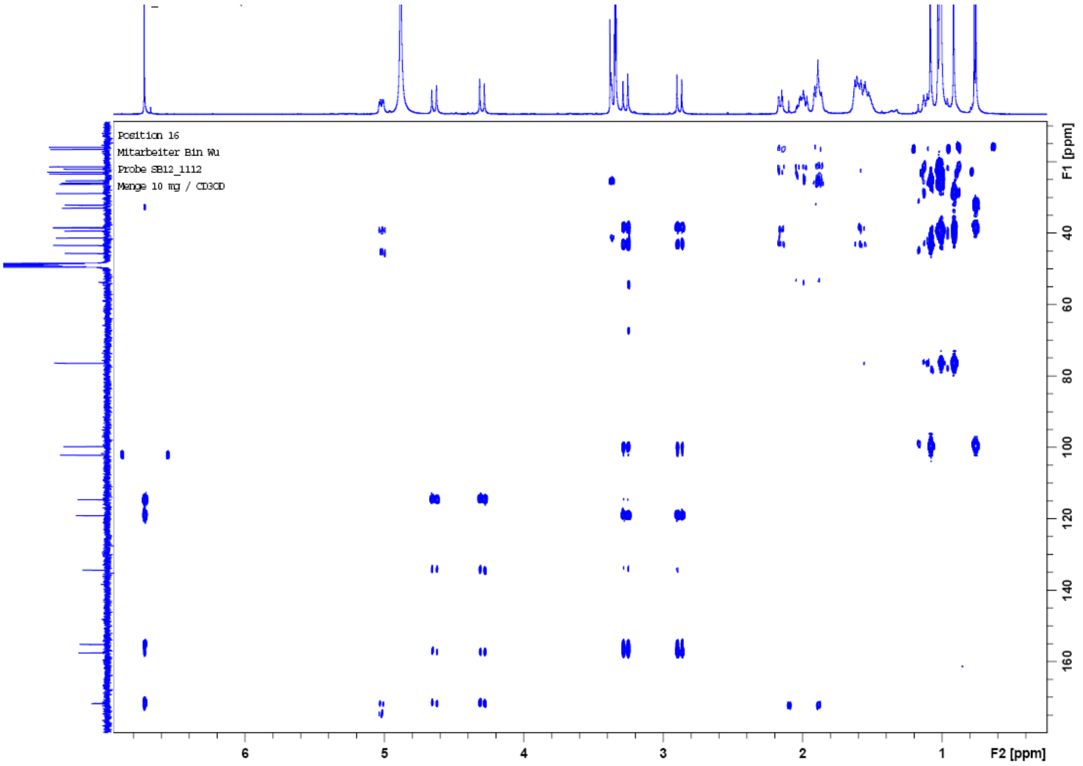

Figure S26. HMBC-2 in CD_3OD for compound **3**.

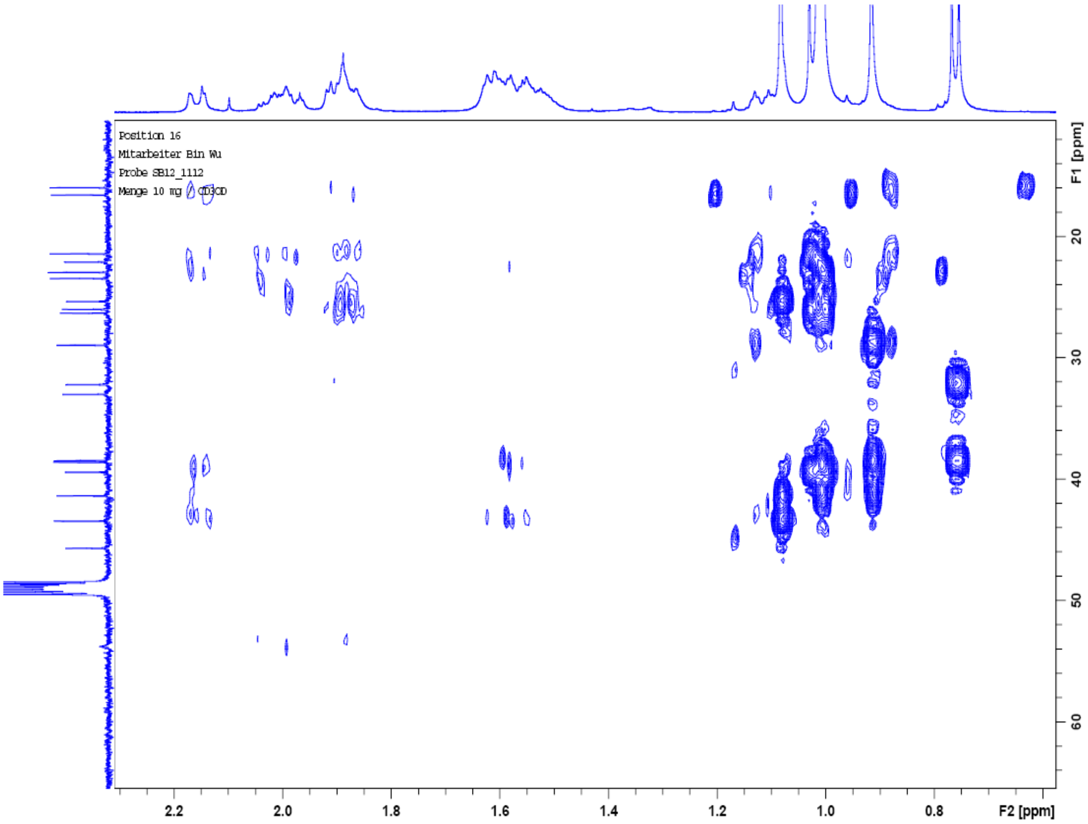

Figure S27. NOESY in CD_3OD for compound 3.

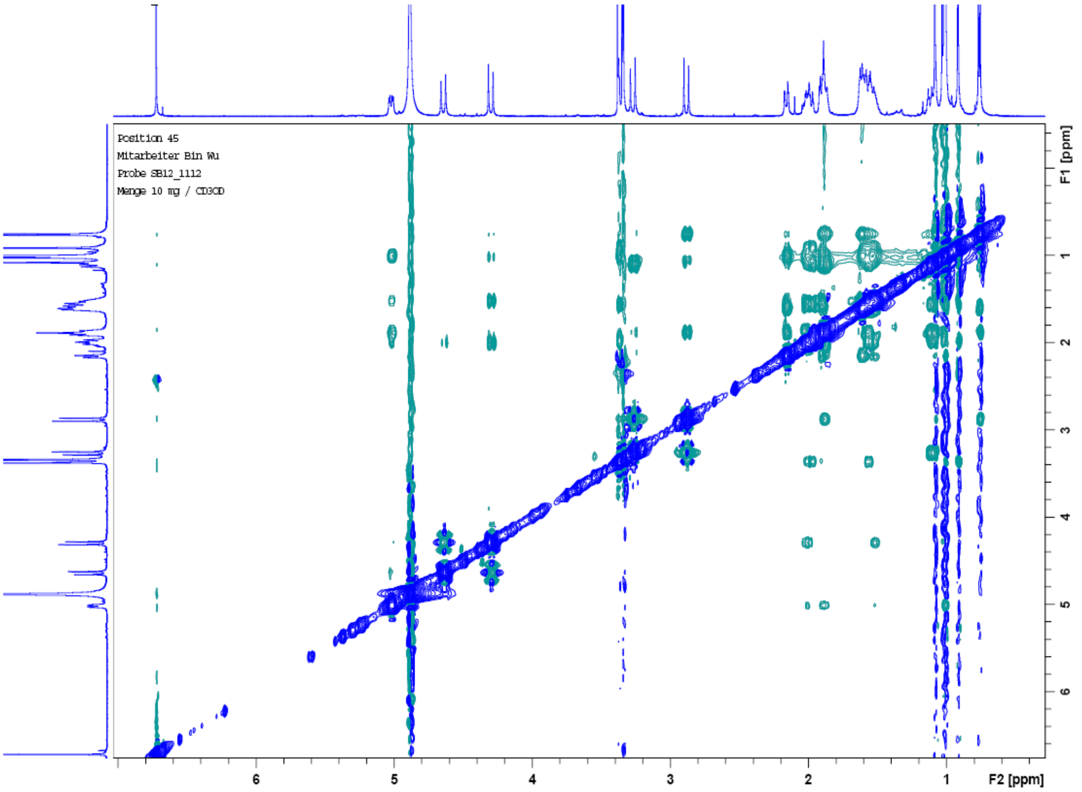

Figure S28. ^1H NMR in CD_3OD for compound 4.

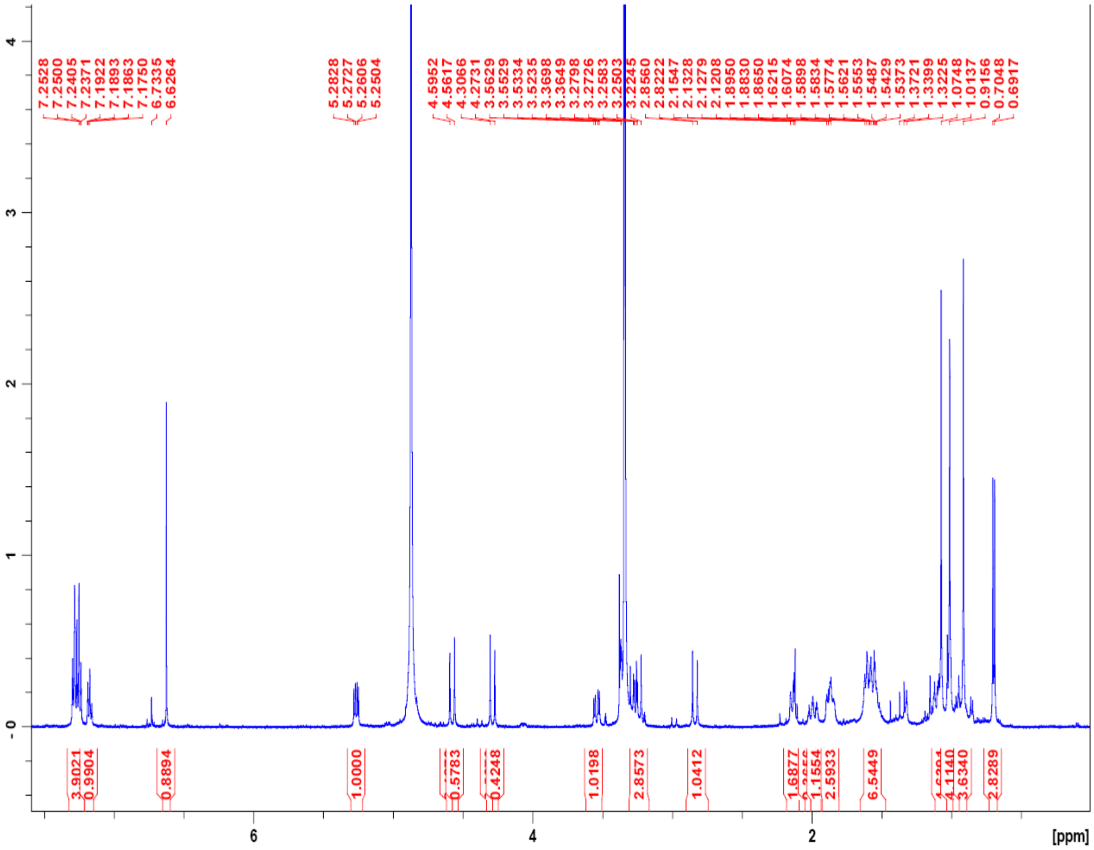

Figure S29. ^{13}C NMR in CD_3OD for compound 4.

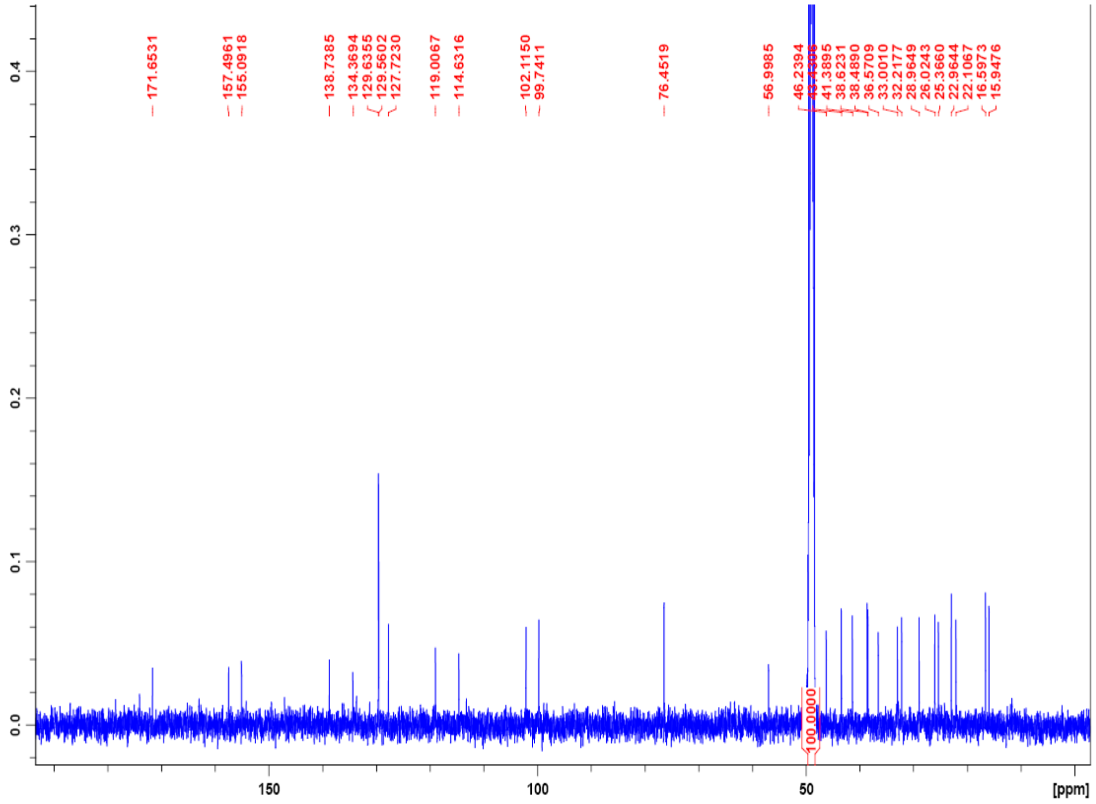

Figure S30. COSY in CD_3OD for compound **4**.

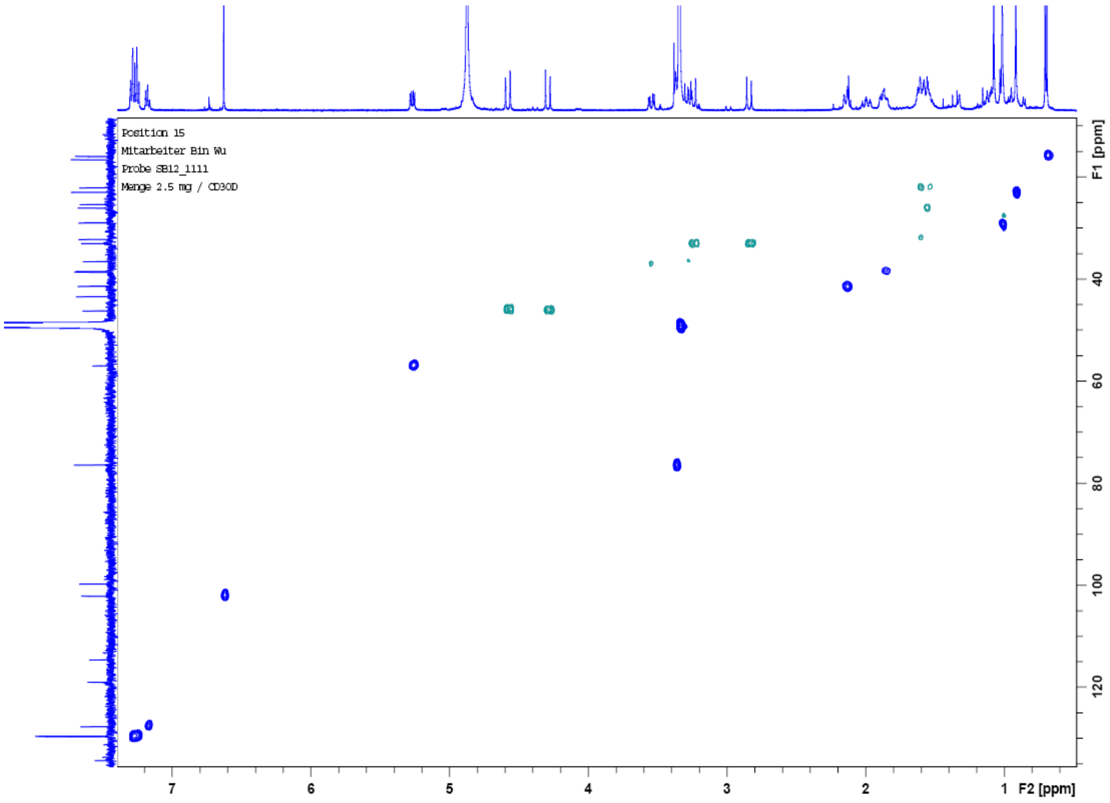

Figure S31. HSQC in CD_3OD for compound **4**.

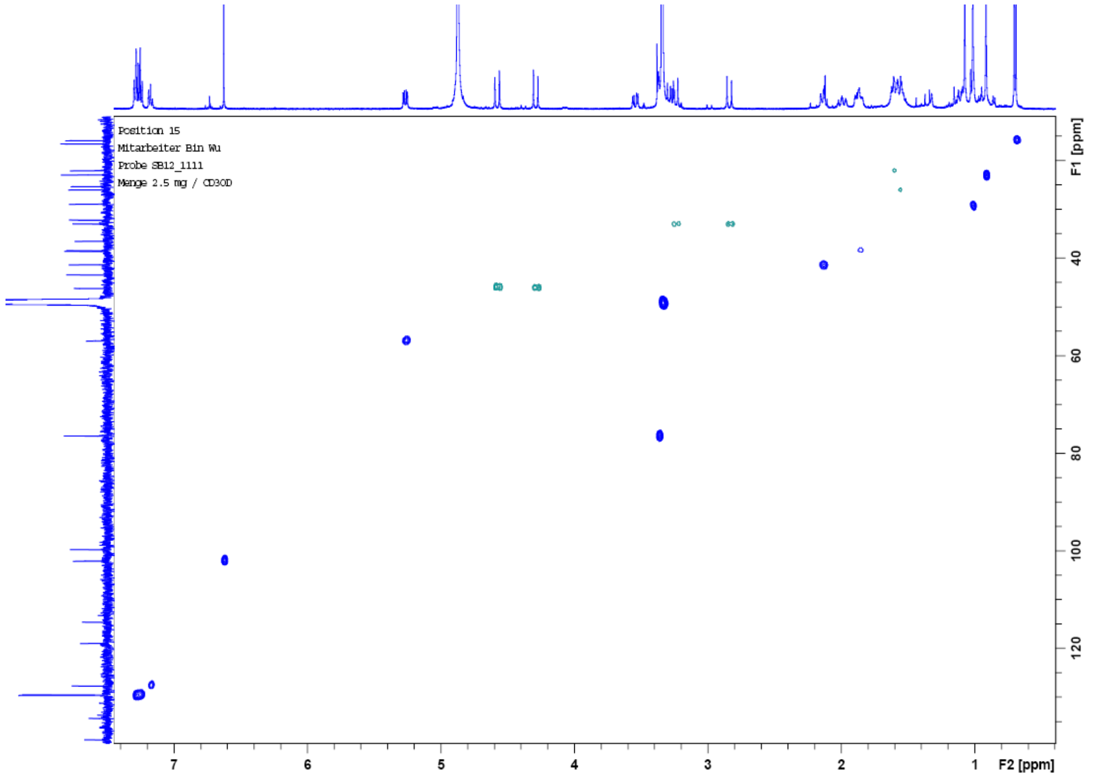

Figure S32. HMBC in CD_3OD for compound **4**.

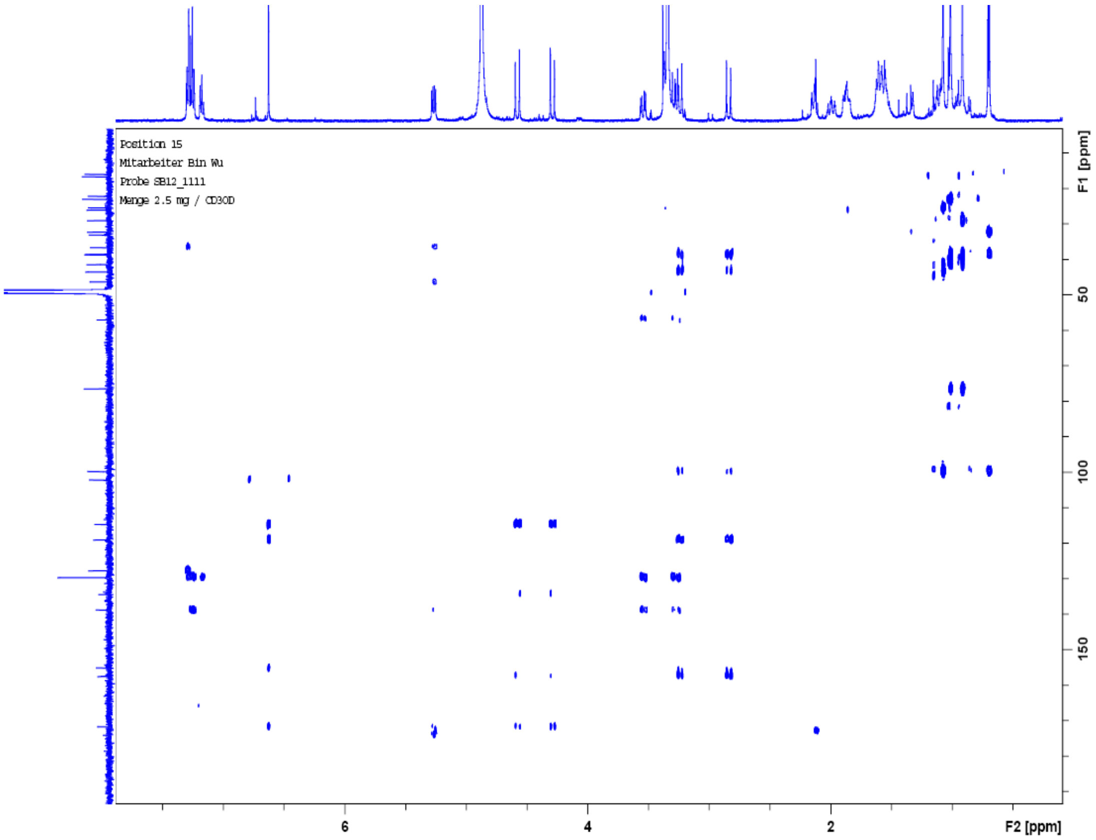

Figure S33. ^{1}H NMR in CD_3OD for compound 5.

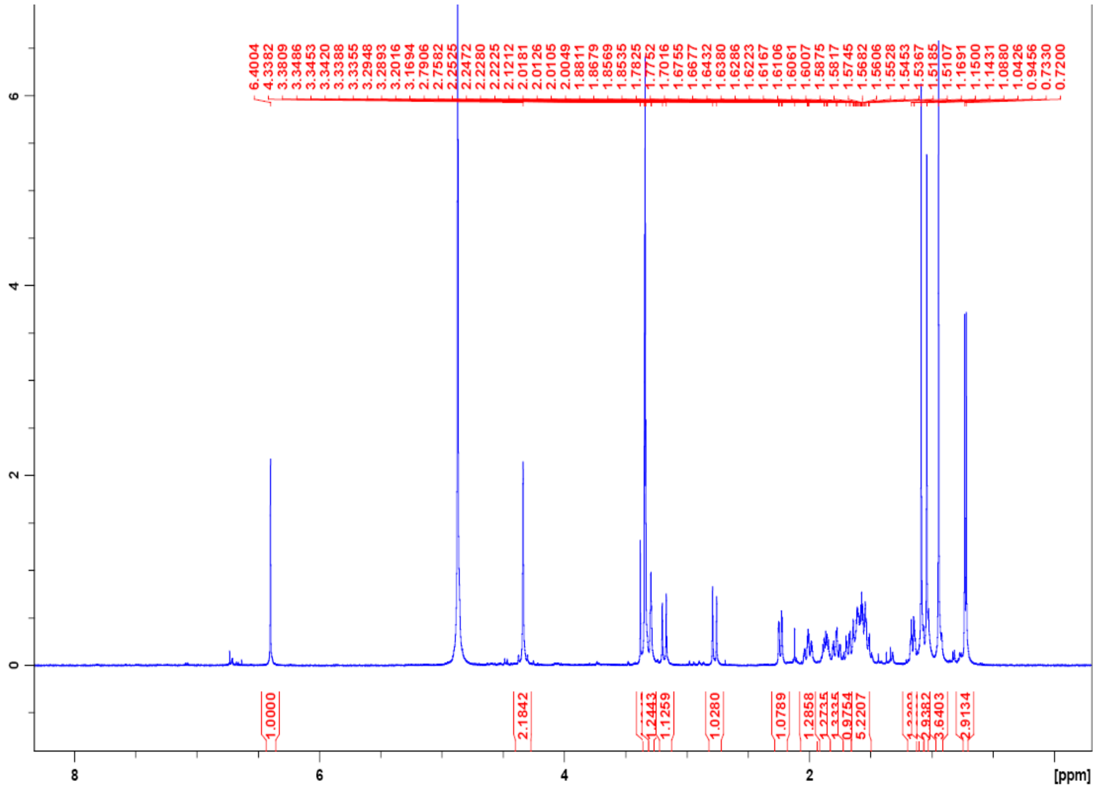

Figure S34. ^{1}H NMR-2 in CD_3OD for compound **5**.

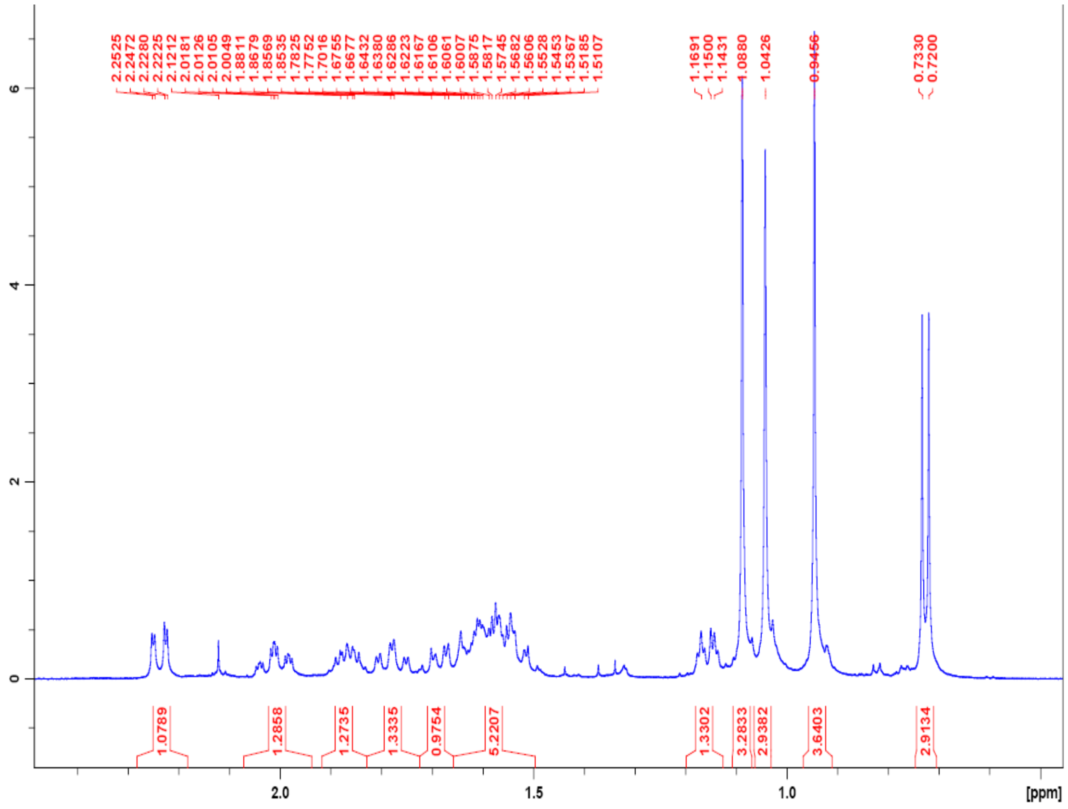

Figure S35. ^{13}C NMR in CD_3OD for compound **5**.

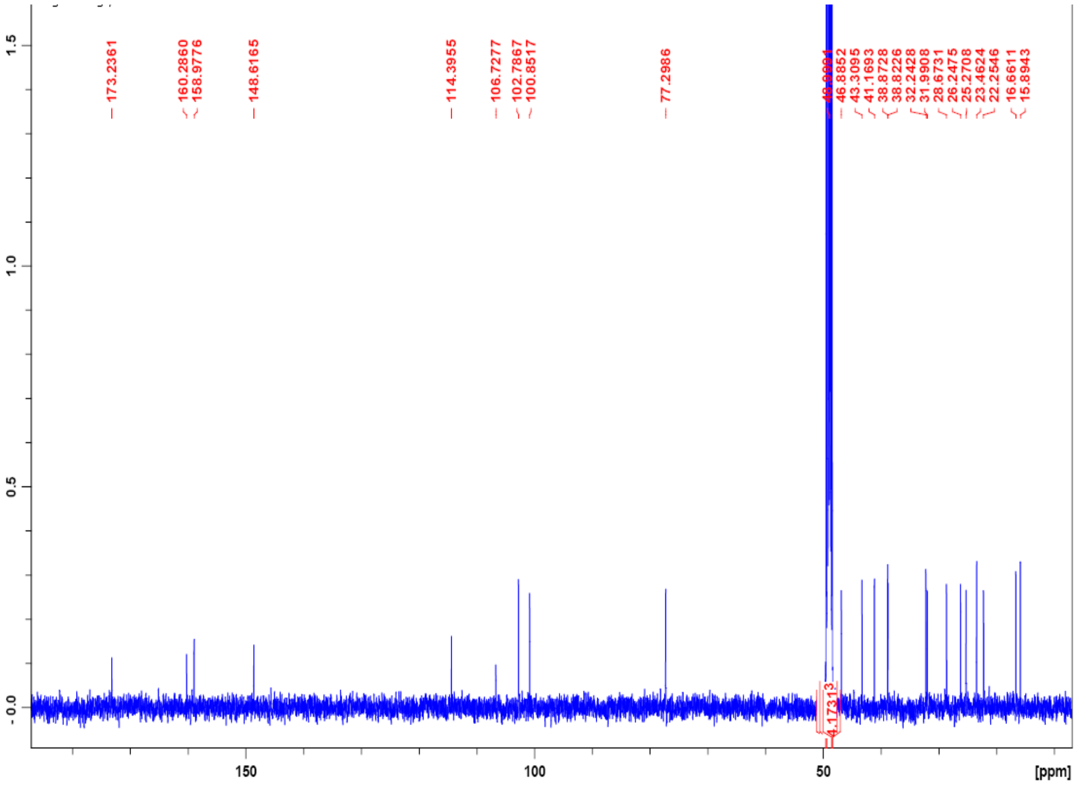

Figure S36. COSY in CD_3OD for compound **5**.

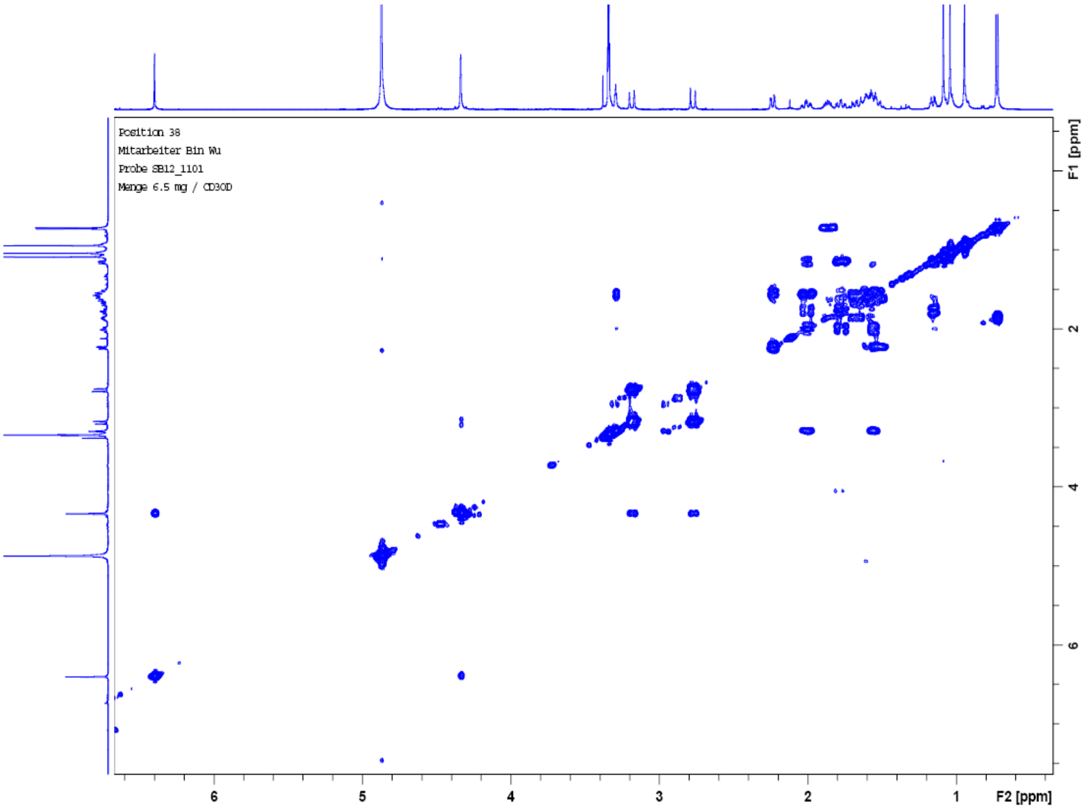

Figure S37. COSY-2 in CD_3OD for compound **5**.

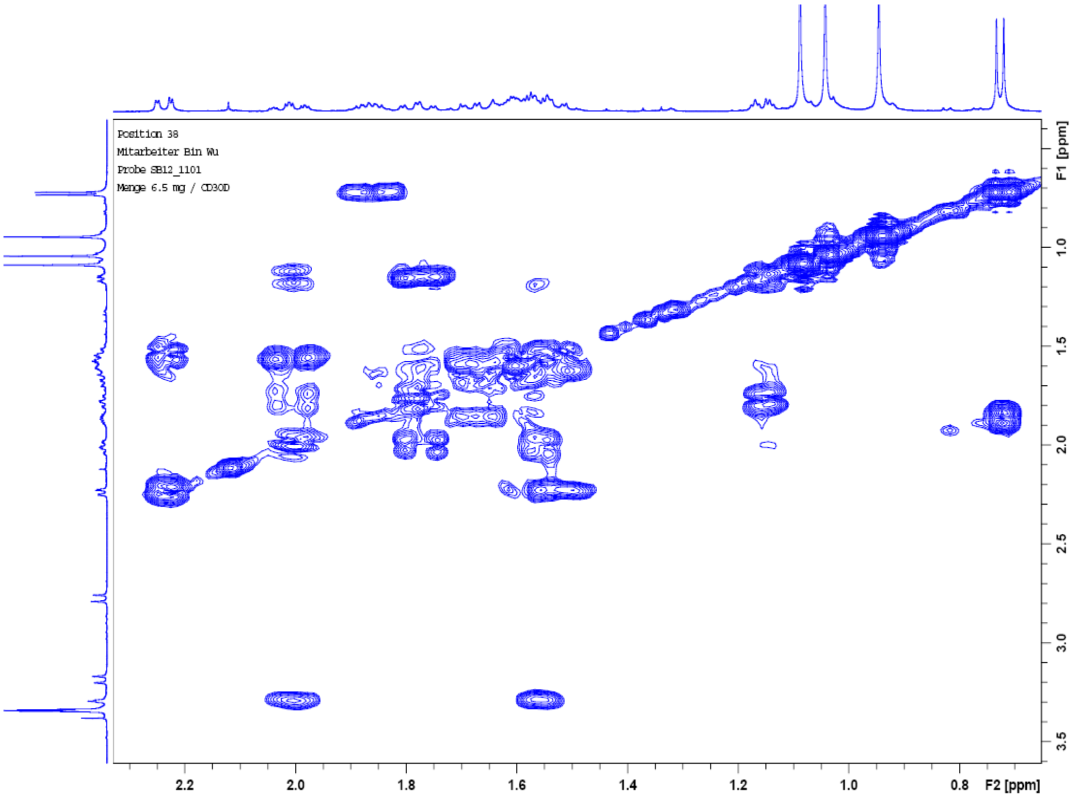

Figure S38. HSQC in CD_3OD for compound **5**.

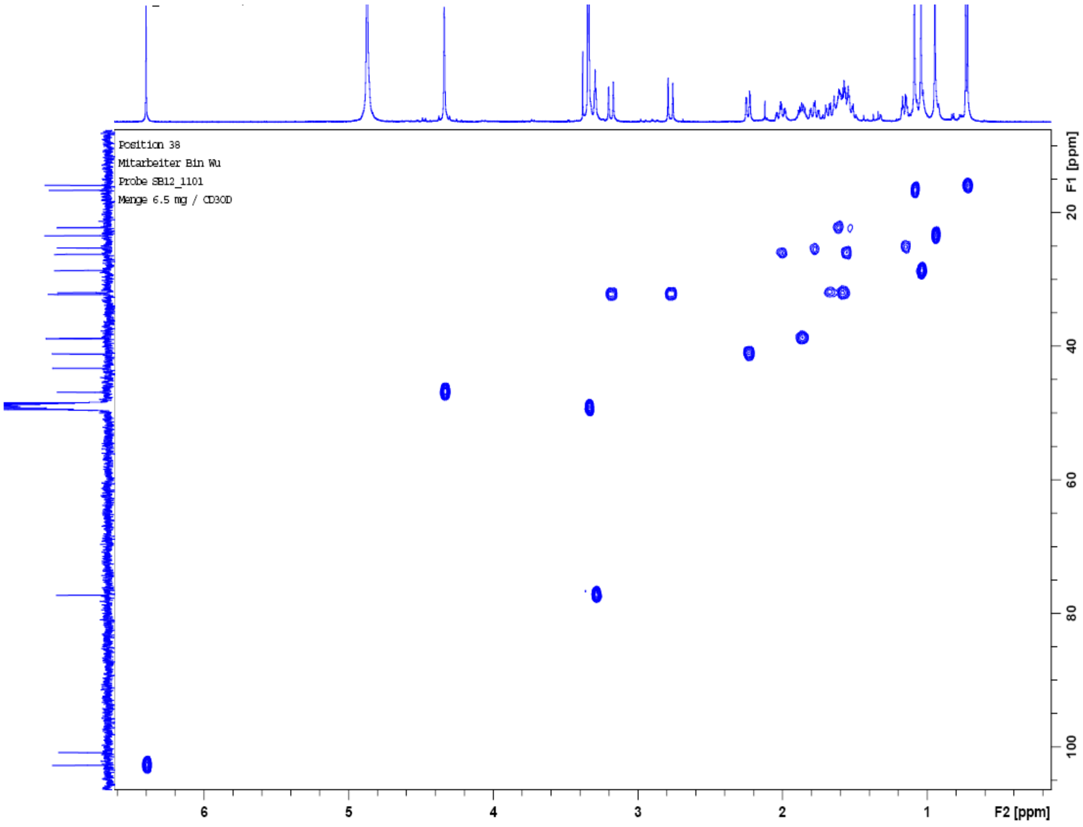

Figure S39. HMBC in CD_3OD for compound **5**.

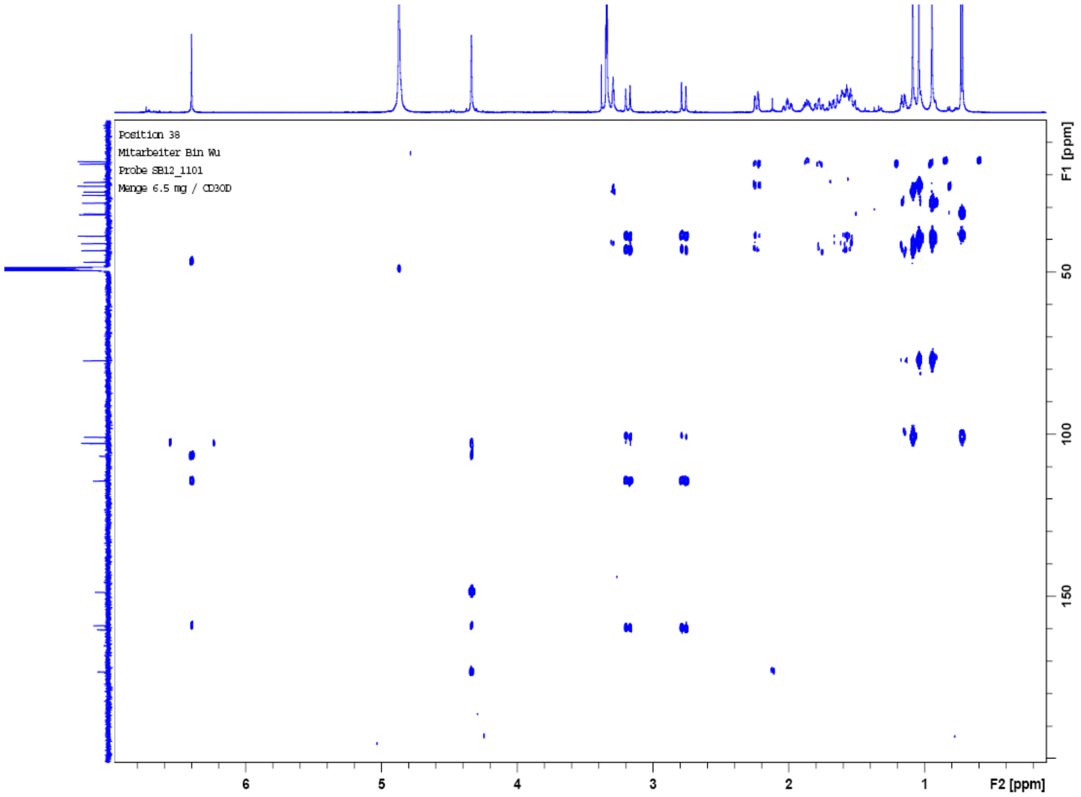

Figure S40. HMBC-2 in CD_3OD for compound **5**.

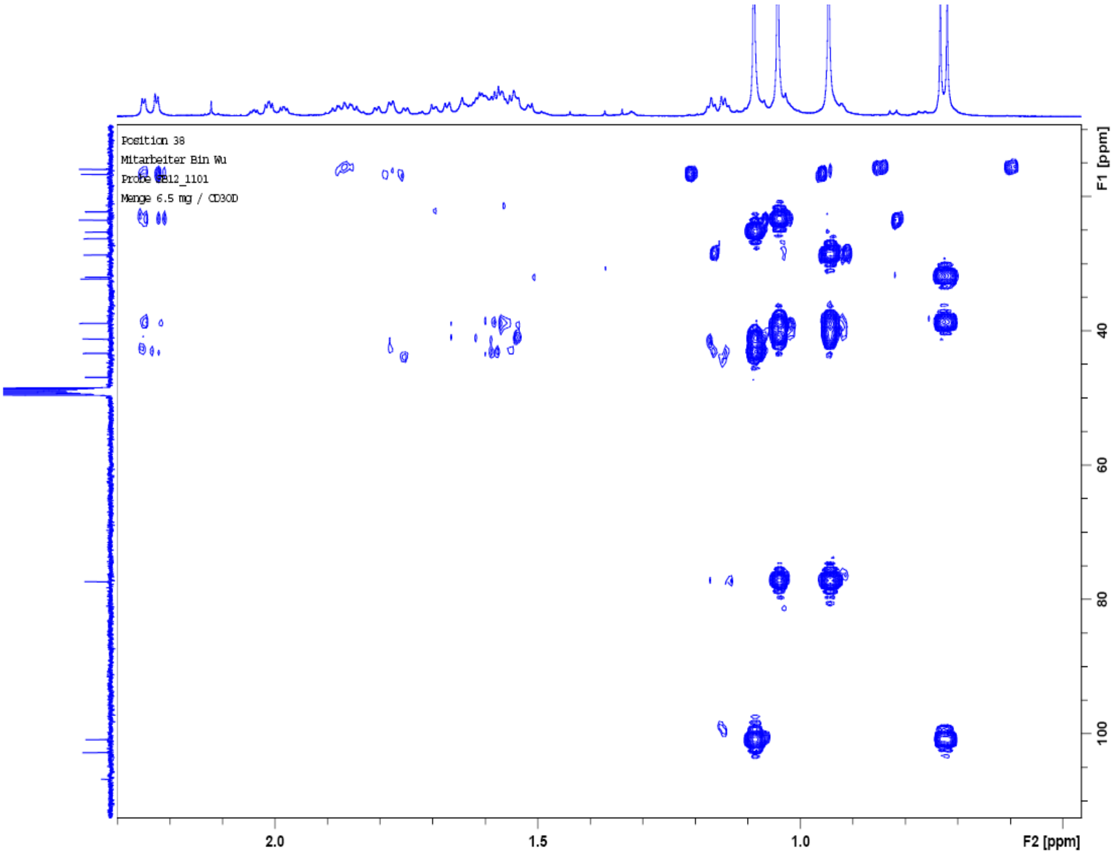

Figure S41. NOESY in CD_3OD for compound **5**.

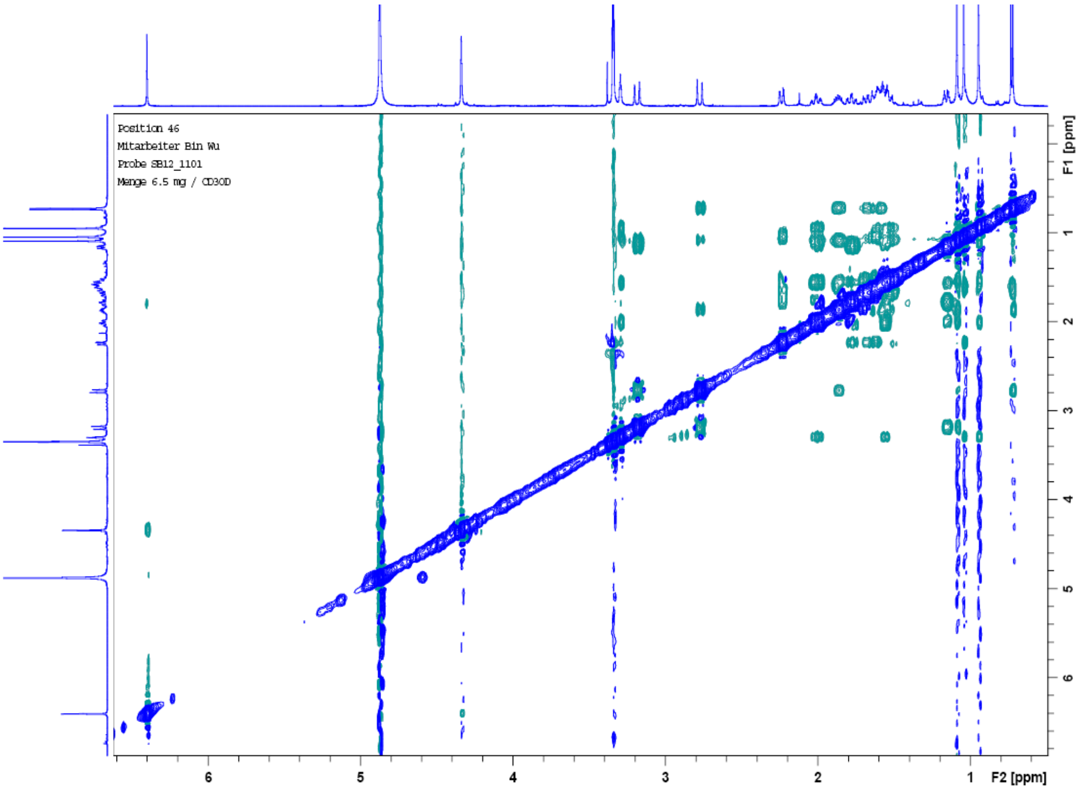

Figure S42. ^{1}H NMR in CD_3OD for compound **6**.

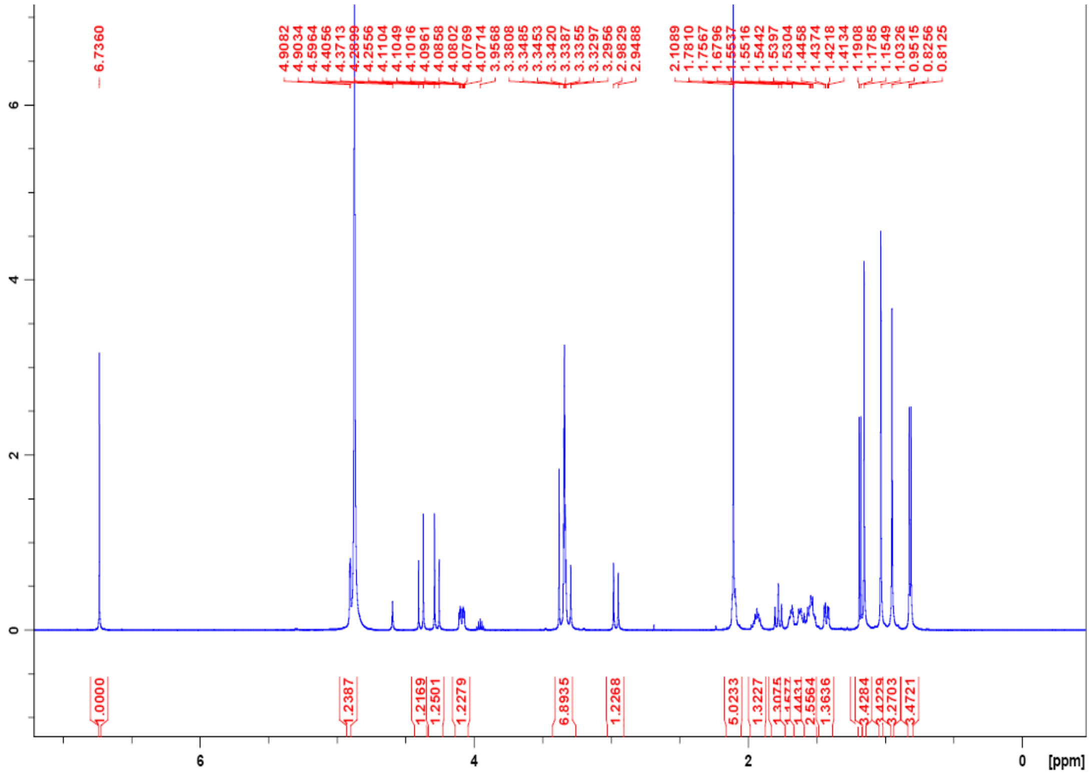

Figure S43. ^{1}H NMR-2 in CD_3OD for compound **6**.

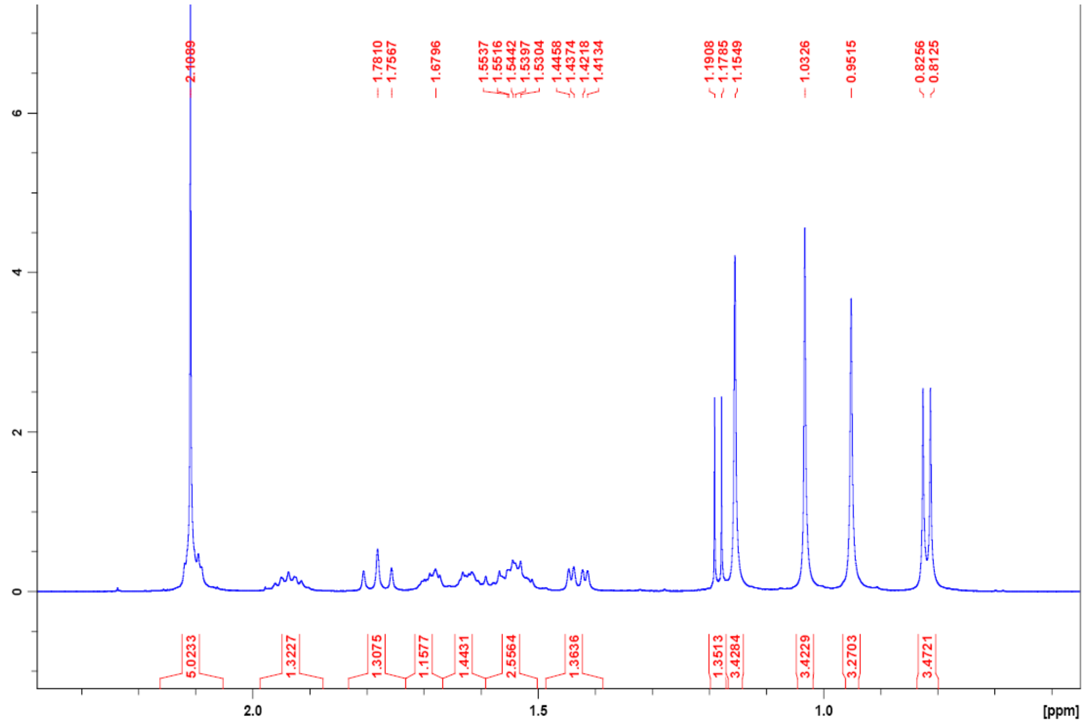

Figure S44. ^{13}C NMR in CD_3OD for compound **6**.

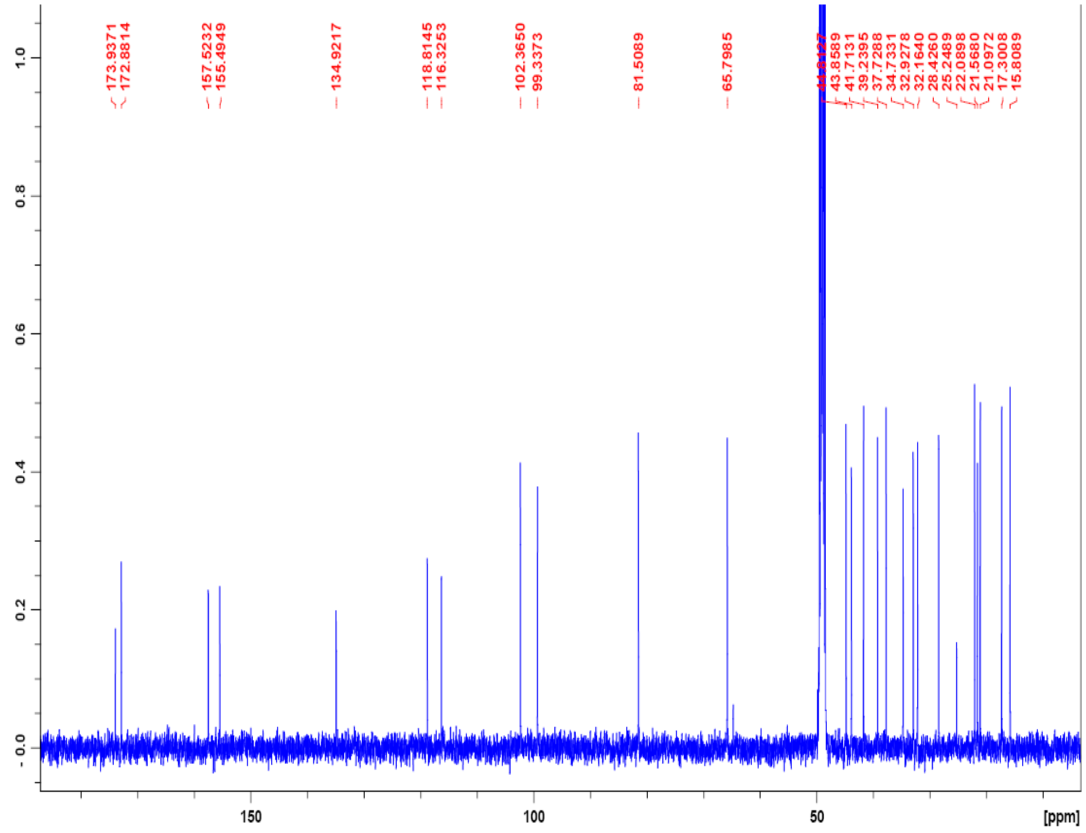

Figure S45. DEPT in CD_3OD for compound **6**.

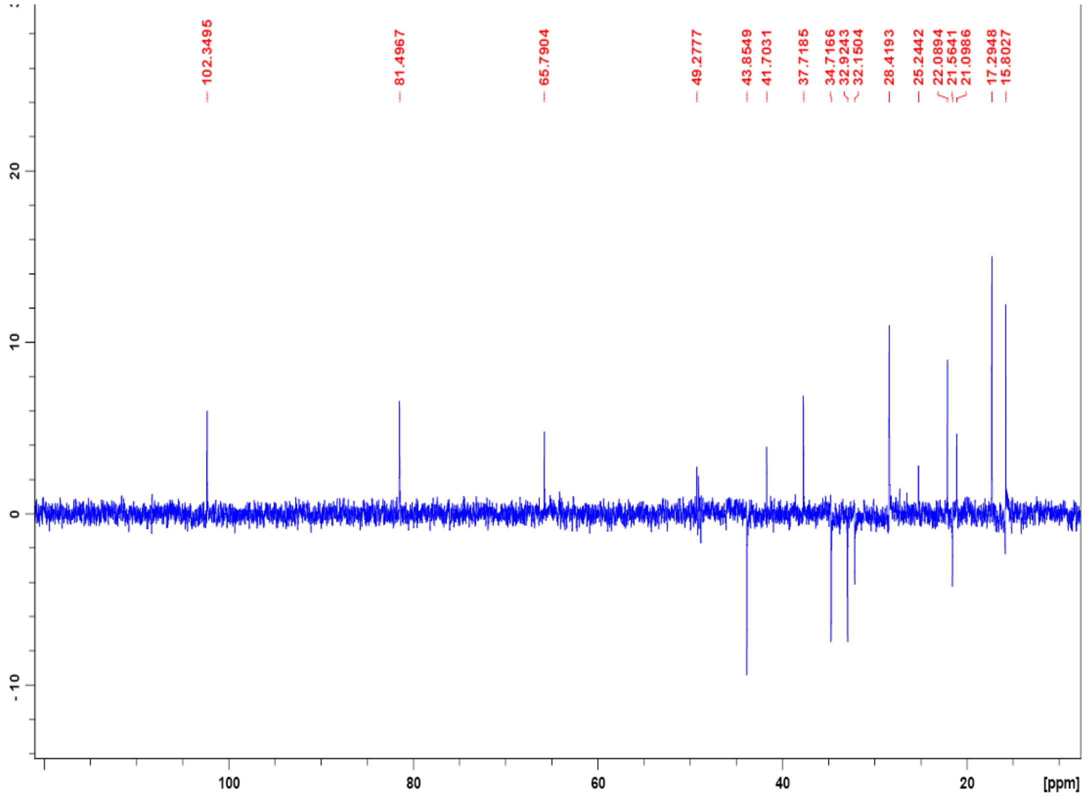

Figure S46. COSY in CD_3OD for compound **6**.

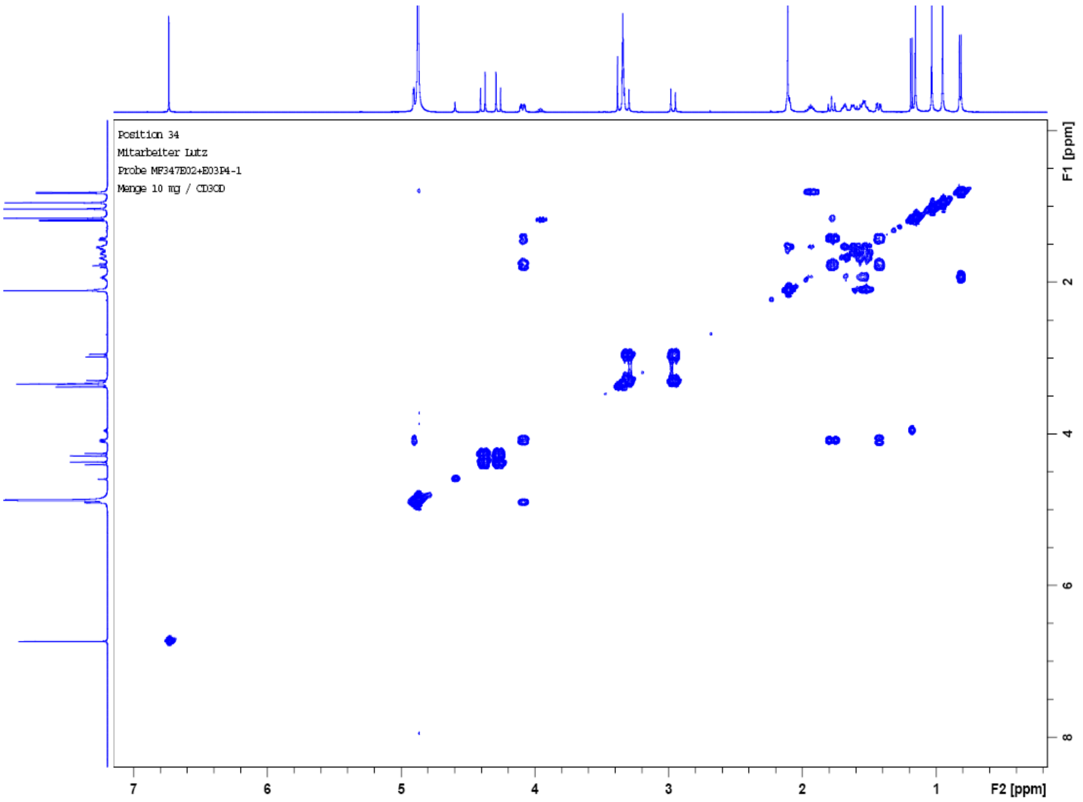

Figure S47. HSQC in CD_3OD for compound **6**.

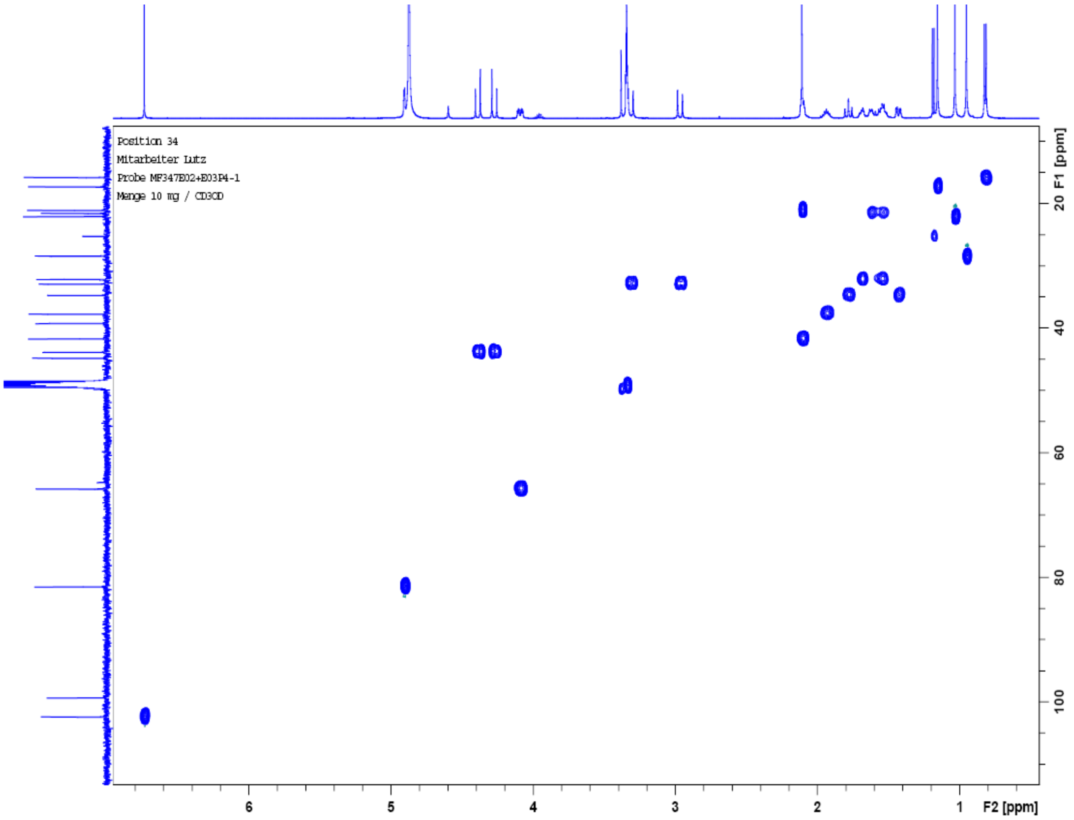

Figure S48. HMBC in CD_3OD for compound **6**.

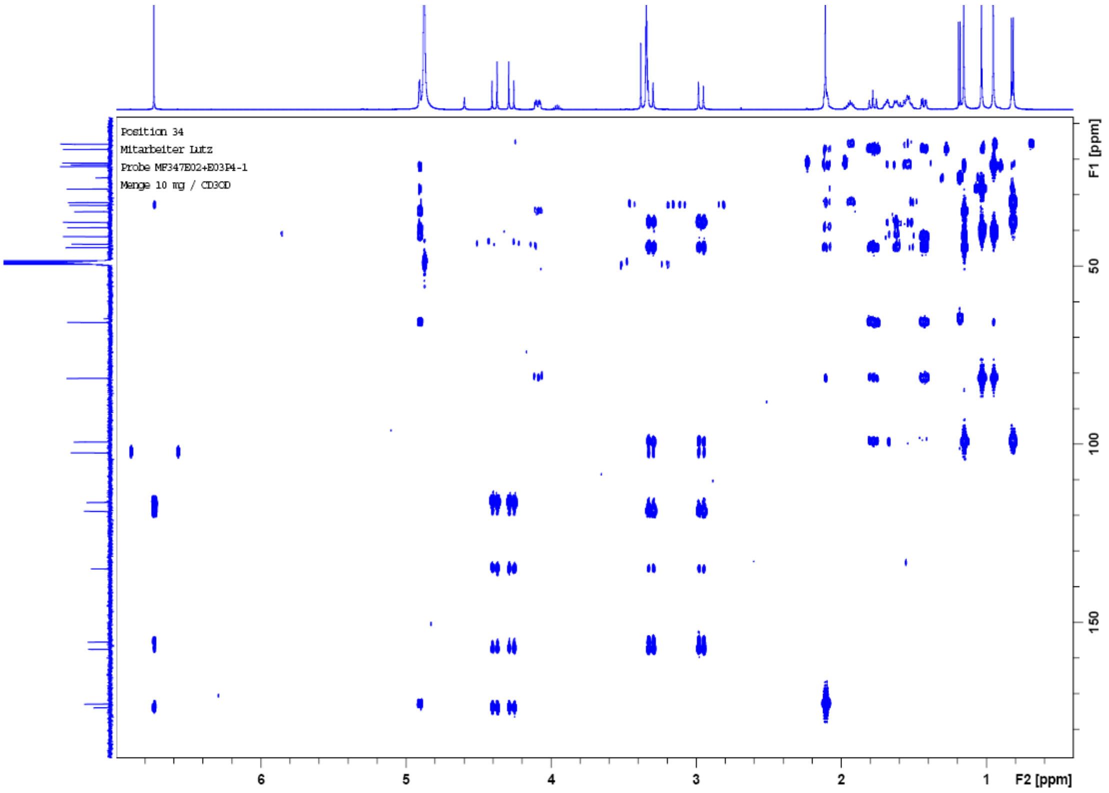

Figure S49. HMBC-2 in CD_3OD for compound 6.

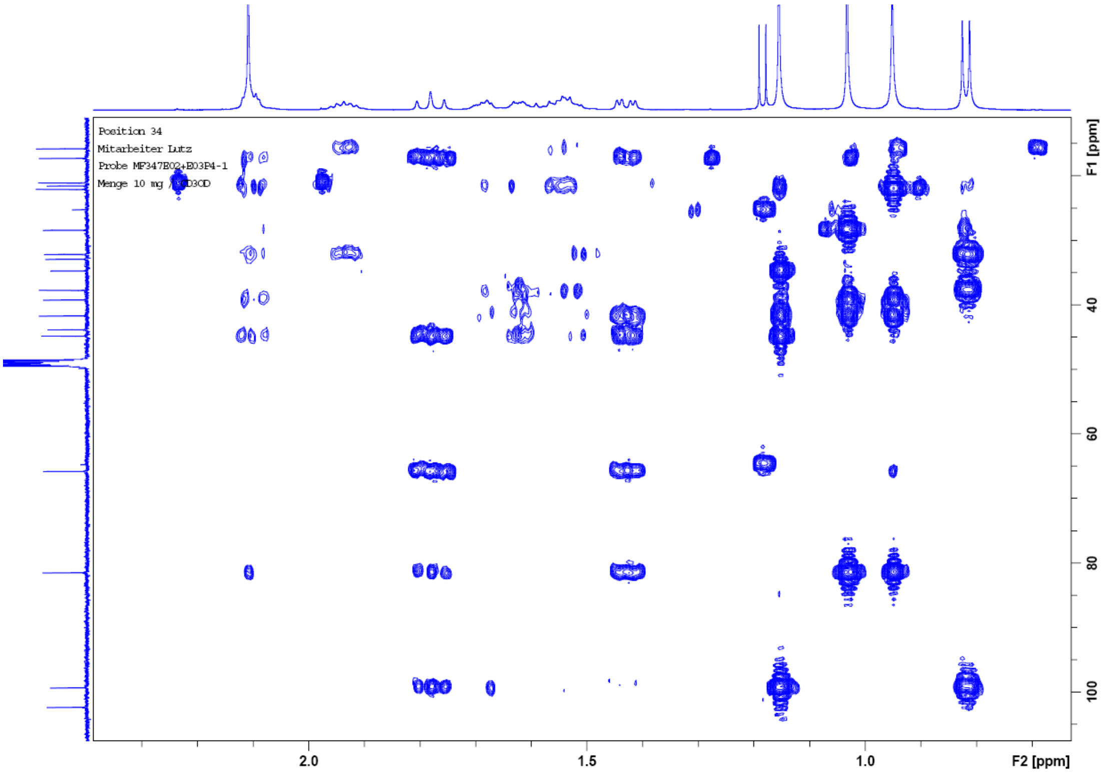

Figure S50. NOESY in CD_3OD for compound **6**.

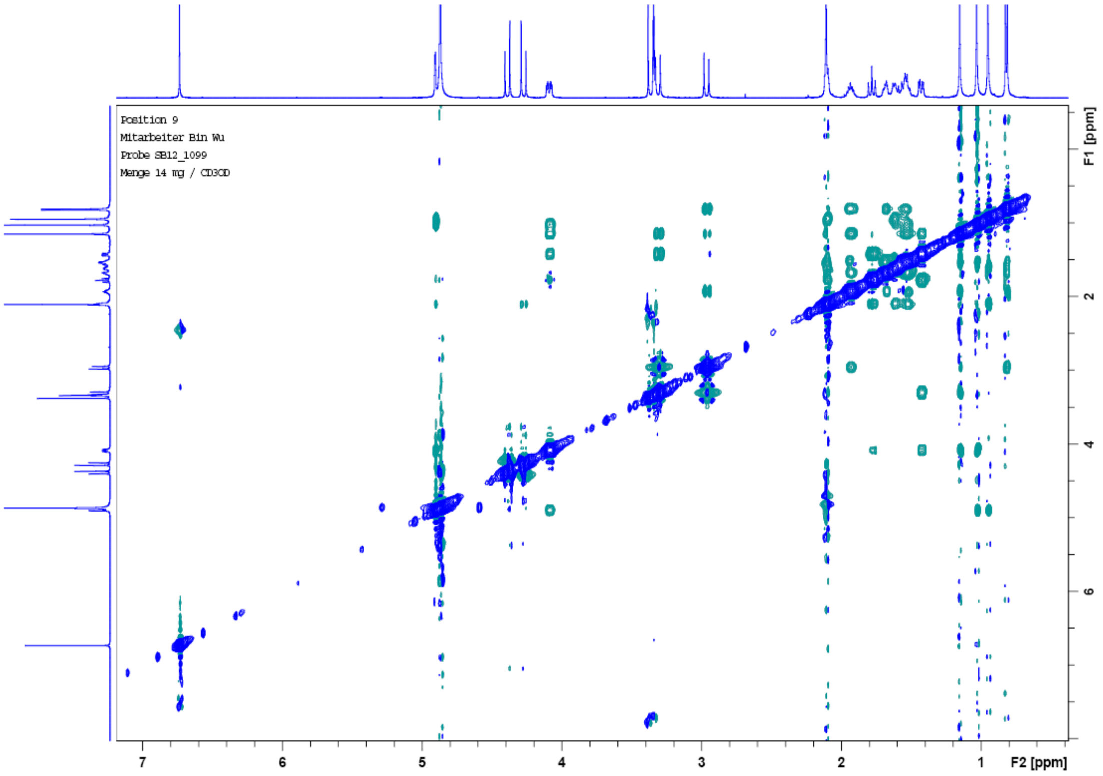

Figure S51. NOESY-2 in CD_3OD for compound **6**.

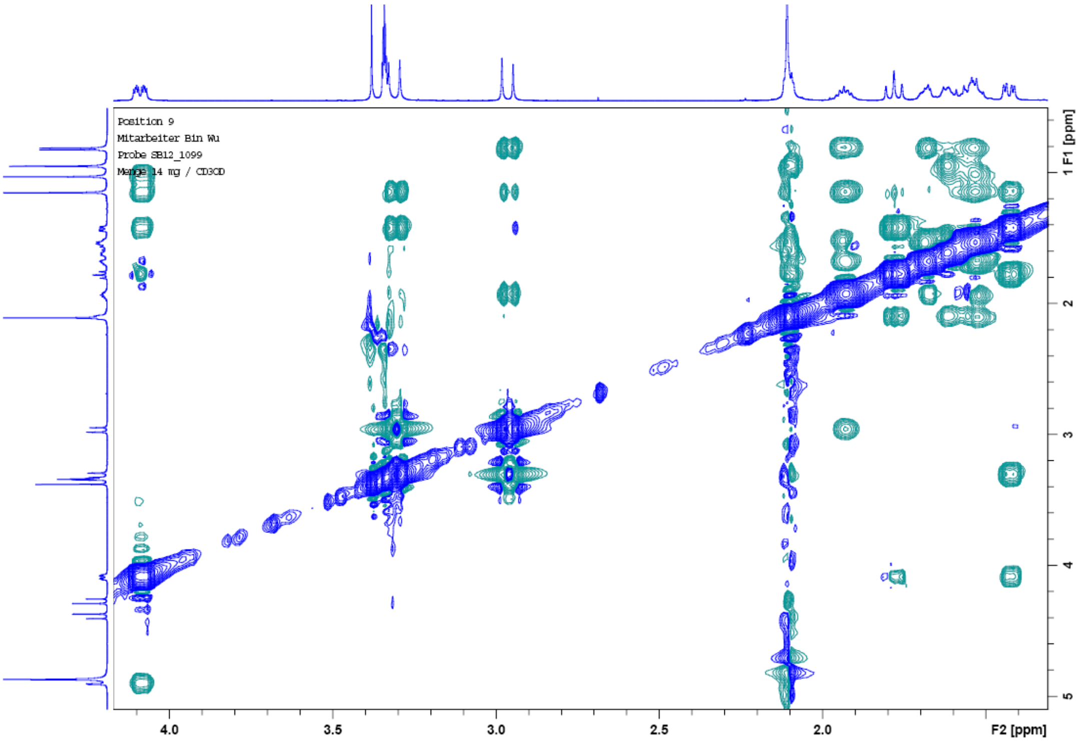

Figure S52. HR-TOF-MS for compound **1**.

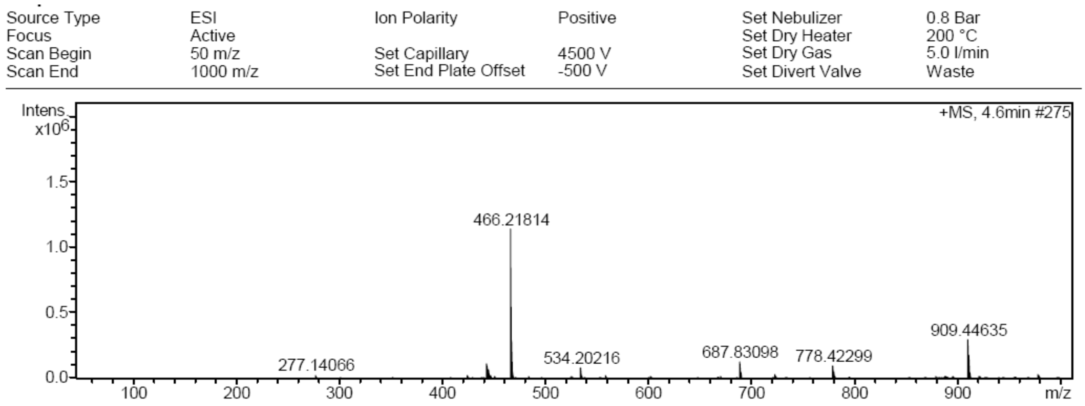

Figure S53. HR-TOF-MS for compound **2**.

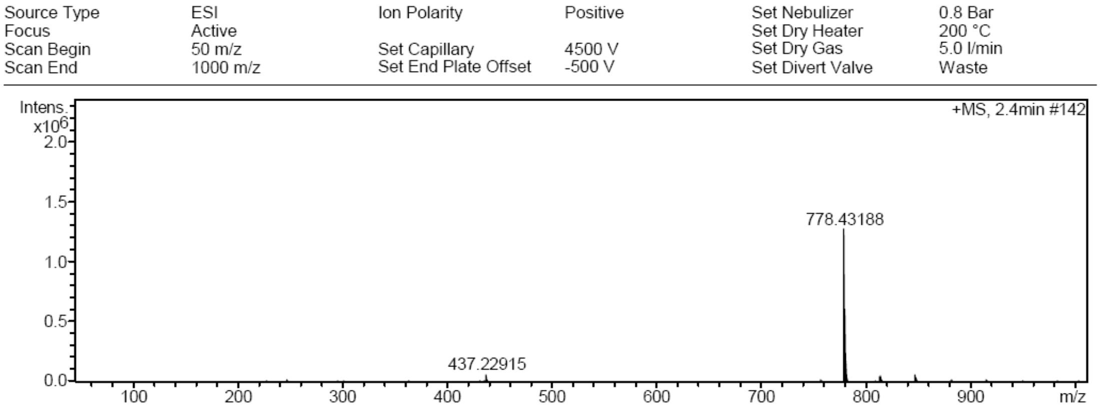

Figure S54. HR-TOF-MS for compound 3.

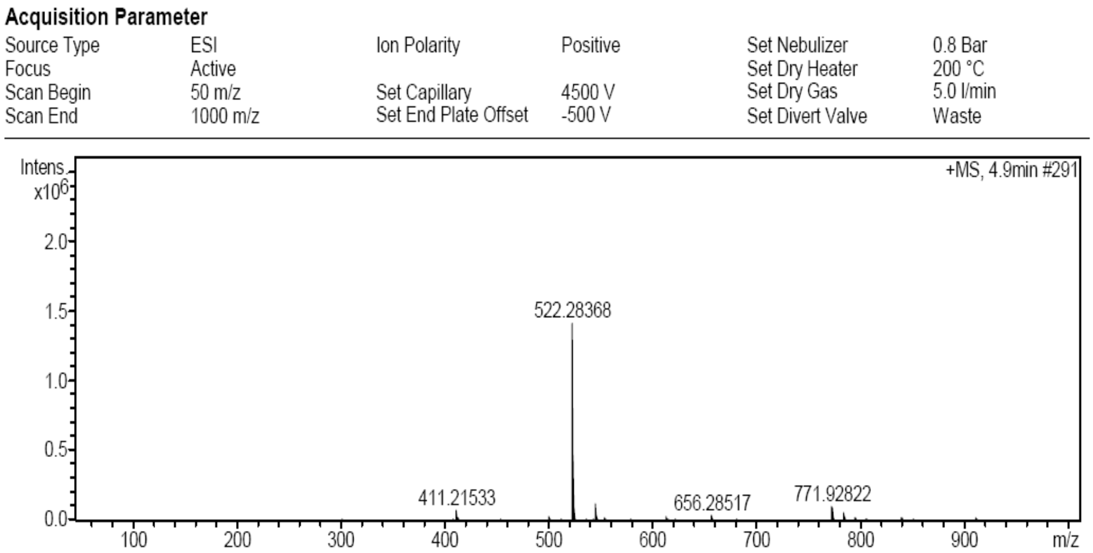

Figure S55. HR-TOF-MS for compound **4**.

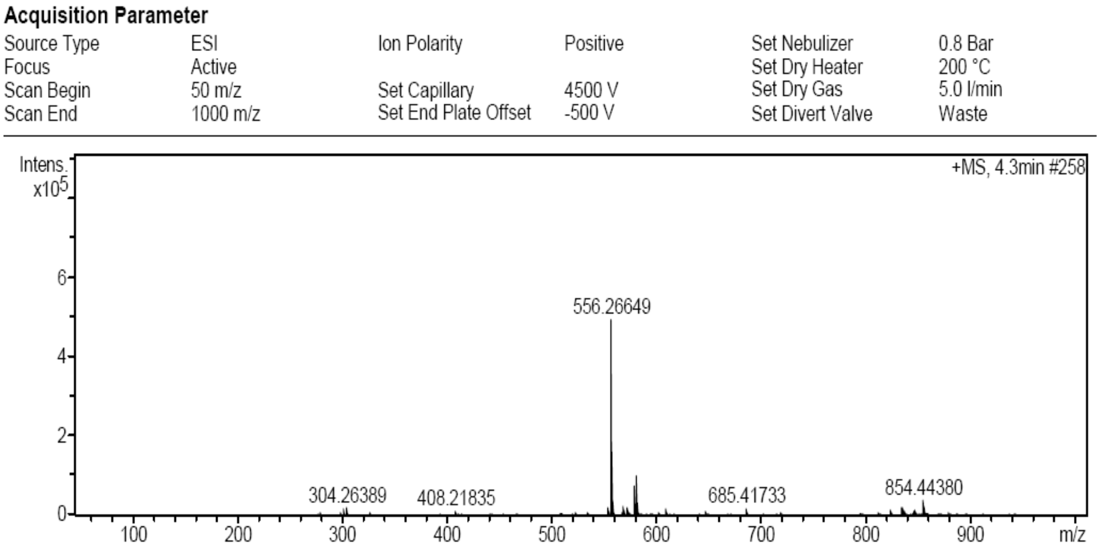

Figure S56. HR-TOF-MS for compound 5.

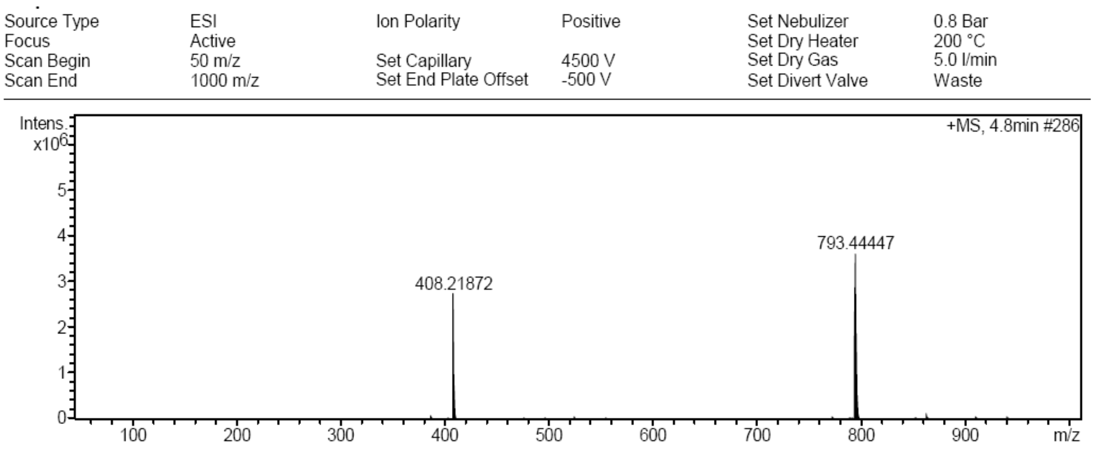

Figure S57. HR-TOF-MS for compound **6**.

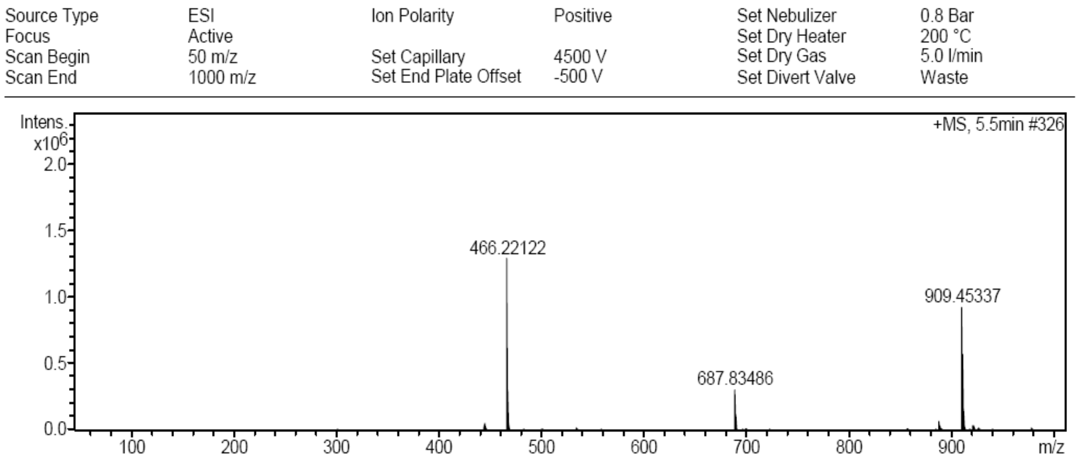

© 2014 by the authors; licensee MDPI, Basel, Switzerland. This article is an open access article distributed under the terms and conditions of the Creative Commons Attribution license (http://creativecommons.org/licenses/by/3.0/).
